# Metabolic independence drives gut microbial colonization and resilience in health and disease

**DOI:** 10.1186/s13059-023-02924-x

**Published:** 2023-04-17

**Authors:** Andrea R. Watson, Jessika Füssel, Iva Veseli, Johanna Zaal DeLongchamp, Marisela Silva, Florian Trigodet, Karen Lolans, Alon Shaiber, Emily Fogarty, Joseph M. Runde, Christopher Quince, Michael K. Yu, Arda Söylev, Hilary G. Morrison, Sonny T. M. Lee, Dina Kao, David T. Rubin, Bana Jabri, Thomas Louie, A. Murat Eren

**Affiliations:** 1 Department of Medicine, The University of Chicago, Chicago, IL 60637 USA; 2 Committee On Microbiology, The University of Chicago, Chicago, IL 60637 USA; 3 Institute for Chemistry and Biology of the Marine Environment, University of Oldenburg, 26129 Oldenburg, Germany; 4 Biophysical Sciences Program, The University of Chicago, Chicago, IL 60637 USA; 5 Department of Medicine, The University of Calgary, Calgary, AB T2N 1N4 Canada; 6 Department of Pediatrics, Lurie Children’s Hospital of Chicago, Chicago, IL 60611 USA; 7 Organisms and Ecosystems, Earlham Institute, Norwich, Norwich, NR4 7UZ UK; 8 Gut Microbes and Health, Quadram Institute, Norwich, NR4 7UQ UK; 9 Toyota Technological Institute at Chicago, Chicago, IL 60637 USA; 10 Department of Computer Engineering, Konya Food and Agriculture University, Konya, Turkey; 11 Marine Biological Laboratory, Josephine Bay Paul Center, Woods Hole, Falmouth, MA 02543 USA; 12 Department of Medicine, University of Alberta, Edmonton, AB T6G 2G3 Canada; 13 Helmholtz Institute for Functional Marine Biodiversity, 26129 Oldenburg, Germany

**Keywords:** Fecal microbiota transplantation, Human gut microbiome, Microbial colonization, Microbial metabolism, Metabolic independence

## Abstract

**Background:**

Changes in microbial community composition as a function of human health and disease states have sparked remarkable interest in the human gut microbiome. However, establishing reproducible insights into the determinants of microbial succession in disease has been a formidable challenge.

**Results:**

Here we use fecal microbiota transplantation (FMT) as an in natura experimental model to investigate the association between metabolic independence and resilience in stressed gut environments. Our genome-resolved metagenomics survey suggests that FMT serves as an environmental filter that favors populations with higher metabolic independence, the genomes of which encode complete metabolic modules to synthesize critical metabolites, including amino acids, nucleotides, and vitamins. Interestingly, we observe higher completion of the same biosynthetic pathways in microbes enriched in IBD patients.

**Conclusions:**

These observations suggest a general mechanism that underlies changes in diversity in perturbed gut environments and reveal taxon-independent markers of “dysbiosis” that may explain why widespread yet typically low-abundance members of healthy gut microbiomes can dominate under inflammatory conditions without any causal association with disease.

**Supplementary Information:**

The online version contains supplementary material available at 10.1186/s13059-023-02924-x.

## Background

Understanding the determinants of microbial colonization is one of the fundamental aims of gut microbial ecology [1, 2]. The gradual maturation of the microbiome during the first months of life [3], the importance of diet and lifestyle in shaping the gut microbiome [4, 5], and the biogeography of microbial populations along the gastrointestinal tract [6] strongly suggest the importance of niche-based interactions between the gut environment and its microbiota. Previous studies that described such interactions in the context of microbial colonization have focused on microbial succession in infant gut microbiomes [3], or relied on model systems such as germ-free mice conventionalized with a consortium of microbial isolates from infant stool [7]. However, our understanding of the ecological underpinnings of secondary succession following a major ecosystem disturbance caused by complex environmental factors in the gut microbiome remains incomplete. A wide range of diseases and disorders are associated with such disturbances, [8–10] however; mechanistic underpinnings of these associations have been difficult to resolve. This is in part due to the diversity of human lifestyles [11], and the limited utility of model systems to make robust causal inferences for microbially mediated human diseases [12].

Inflammatory bowel disease (IBD), a group of increasingly common intestinal disorders that cause inflammation of the gastrointestinal tract [13], has been a model to study human diseases associated with the gut microbiota [14]. The pathogenesis of IBD is attributed in part to the gut microbiome [15], yet the microbial ecology of IBD-associated dysbiosis remains a puzzle. Despite marked changes in gut microbial community composition in IBD [16–18], the microbiota associated with the disease lacks acquired infectious pathogens [19], and microbes that are found in IBD typically also occur in healthy individuals [20], which complicates the search for robust functional or taxonomic markers of health and disease states [21]. One of the hallmarks of IBD is reduced microbial diversity during episodes of inflammation, when the gut environment is often dominated by microbes that typically occur in lower abundances prior to inflammation [22]. The sudden increase in the relative abundance of microbes that are also common to healthy individuals suggests that the harsh conditions of IBD likely act as an ecological filter that eliminates some populations while allowing others to bloom. Yet, in the absence of an understanding of the genetic requirements for survival in IBD, critical insights into the functional drivers of microbial community succession in such disease states remains elusive.

Fecal microbiota transplantation (FMT), the transfer of stool from a donor into a recipient’s gastrointestinal tract [23], represents an experimental middleground to capture complex ecological interactions that shape the microbial community during secondary succession of a disrupted gut environment. FMT is frequently employed in the treatment of recurrent *Clostridioides difficile* infection (CDI) [24] that can cause severe diarrhea and intestinal inflammation. In addition to its medical utility, FMT offers a powerful framework to study fundamental questions of microbial ecology by colliding the microbiome of a healthy donor with the disrupted gut environment of the recipient. The process presents an ecological filter with the potential to reveal functional determinants of microbial colonization success and resilience in impaired gut environments [25].

Here we use FMT as an in natura experimental model to investigate the ecological and functional determinants of successful colonization of the human gut at the level of individual microbial populations using genome-resolved metagenomics. Our findings highlight the importance of environmental selection acting on the biosynthetic capacity for essential nutrients as a key driver of not only colonization outcomes after FMT but also microbial resilience during inflammation, and demonstrate that 'metabolic independence' can serve as a taxonomy-independent determinant of colonization success in the human gut under stress.

## Results and discussion

### Study design

**Fig. 1:**
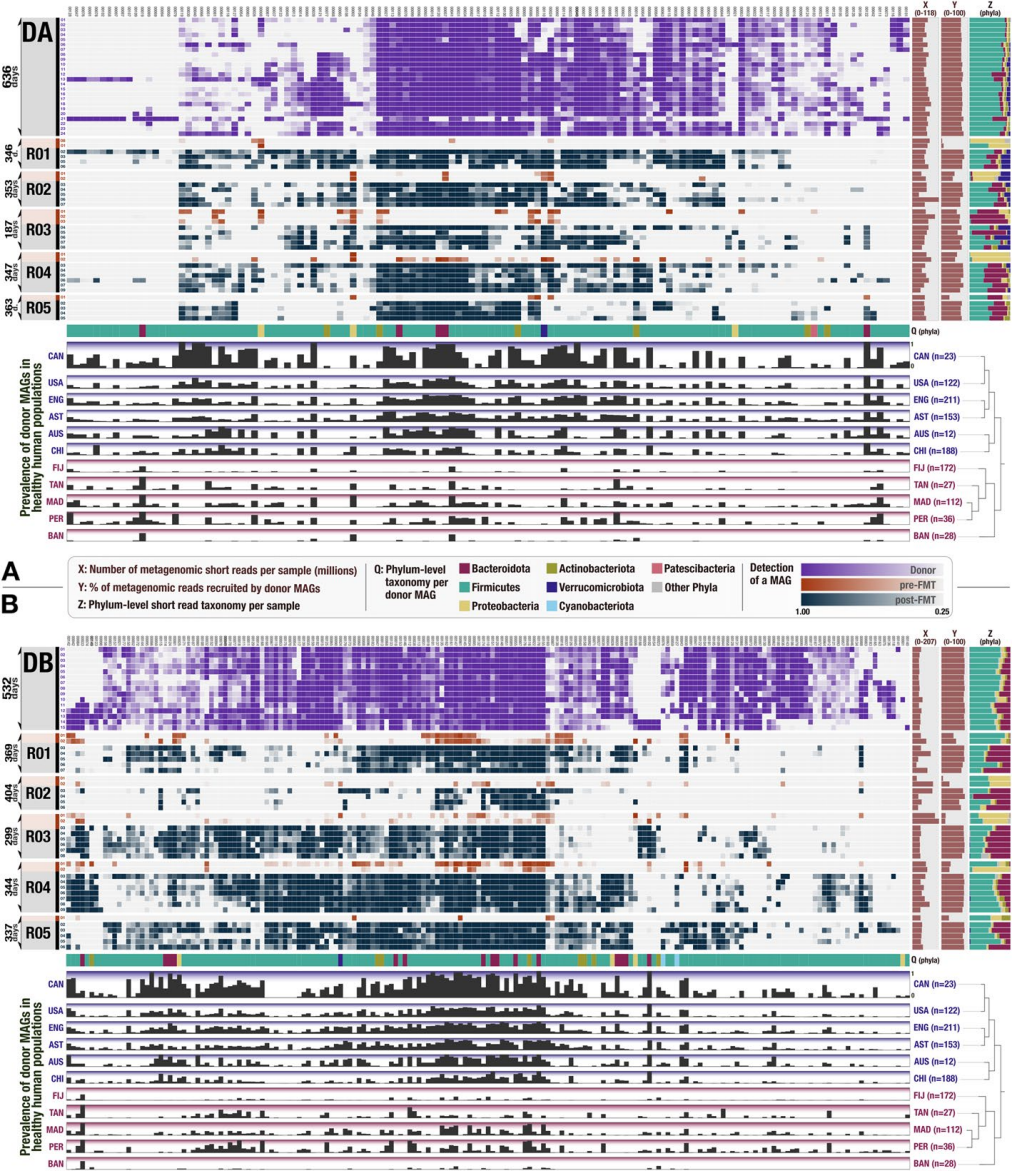
Detection of FMT donor genomes in FMT recipients and publicly available gut metagenomes. In both heat maps, each column represents a donor genome, each row represents a metagenome, and each data point represents the detection of a given genome in a given metagenome. Purple rows represent donor metagenomes from stool samples collected over 636 days for **A** donor A and 532 days for **B** donor B. Orange rows represent recipient pre-FMT metagenomes, and blue rows represent recipient post-FMT metagenomes. Rows are arranged in descending chronological order with respect to each subject. The intensity of purple, orange, and blue color scales represents the detection value for each genome in each metagenome, with a minimum detection of 0.25. Genome columns are clustered according to their presence or absence in all metagenomes (Euclidean distance and Ward clustering). The three columns to the right of the heatmaps display, for each metagenome row: (X) the number of metagenomic short reads in millions, (Y) the percent of metagenomic short reads recruited by genomes, and (Z) the taxonomic composition of metagenomes (based on metagenomic short reads) at the phylum level. The first row below each heat map (Q) provides the phylum-level taxonomy for each donor genome. Finally, the 11 bottommost rows under each heat map show the fraction of healthy adult metagenomes from 11 different countries in which a given donor genome is detected (if a genome is detected in every individual from a country it is represented with a full bar and a value of 1). The dendrograms on the right-hand side of the country layers organize countries based on the detection patterns of genomes (Euclidean distance and Ward clustering). Purple and red shaded countries represent the two main clusters that emerge from this analysis, where purple layers are industrialized countries in which donor genomes are highly prevalent and red layers are less industrialized countries where the prevalence of donor genomes is low. A maximum resolution version of this figure is also available at https://doi.org/10.6084/m9.figshare.15138720 [26]

Our study includes 109 gut metagenomes (Additional file 1) from two healthy FMT donors (A and B) and 10 FMT recipients (five recipients per donor) with multiple recurrent CDI. We collected 24 donor A samples over a period of 636 days and 15 donor B samples over a period of 532 days to establish an understanding of the long-term microbial population dynamics within each donor microbiota. The FMT recipients received vancomycin for a minimum of 10 days to attain resolution of diarrheal illness prior to FMT. On the last day of vancomycin treatment, a baseline fecal sample was collected from each recipient, and their bowel contents were evacuated immediately prior to FMT. Recipients did not take any antibiotics on the day of transplant, or during the post-FMT sampling period (Additional file 2: Fig. S1). We collected 5 to 9 samples from each recipient for a period of up to 336 days post-FMT. Deep sequencing of donor and recipient metagenomes using Illumina paired-end (2 × 150) technology resulted in a total of 7.7 billion sequences with an average of 71 million reads per metagenome (Fig. 1, Additional file 1, Additional file 3). We employed genome-resolved metagenomics, microbial population genetics, and metabolic pathway reconstruction for an in-depth characterization of donor and recipient gut microbiotas, and we leveraged publicly available gut metagenomes to benchmark our observations.

### Genome-resolved metagenomics show many, but not all, donor microbes colonized recipients and persisted long-term

We first characterized the taxonomic composition of each donor and recipient sample by analyzing our metagenomic short reads given a clade-specific k-mer database (Additional file 3). The phylum-level microbial community composition of both donors reflected those observed in healthy individuals in North America [27]: a large representation of Firmicutes and Bacteroidetes, and other taxa with lower relative abundances including Actinobacteria, Verrucomicrobia, and Proteobacteria (Fig. 1, Additional file 3). In contrast, the vast majority of the recipient pre-FMT samples were dominated by Proteobacteria, a phylum that typically undergoes a drastic expansion in individuals treated with vancomycin [28]. After FMT, we observed a dramatic shift in recipient taxonomic profiles (Additional file 3, Additional file 2: Fig. S2, Additional file 2: Fig. S3), a widely documented hallmark of this procedure [29–31]. Nearly all recipient samples post-FMT were dominated by Bacteroidetes and Firmicutes as well as Actinobacteria and Verrucomicrobia in lower abundances, resembling qualitatively, but not quantitatively, the taxonomic profiles of their donors (Additional file 3). The phylum Bacteroidetes was over-represented in recipients: even though the median relative abundance of Bacteroidetes populations were 5 and 17% in donors A and B, their relative abundance in recipients post-FMT was 33 and 45%, respectively (Fig. 1, Additional file 3). A single genus, *Bacteroides*, made up 76 and 82% of the Bacteroidetes populations in the recipients of donor A and B, respectively (Additional file 3). The success of the donor *Bacteroides* populations in recipients upon FMT is not surprising given the ubiquity of this genus across geographically diverse human populations [32] and the ability of its members to survive substantial levels of stress [22, 33]. This initial coarse taxonomic analysis demonstrates the successful transfer of only some populations, suggesting selective filtering of the transferred community.

To generate insights into the genomic content of the microbial community, we first assembled short metagenomic reads into contiguous segments of DNA (contigs). Co-assemblies of 24 donor A and 15 donor B metagenomes independently resulted in 53,891 and 54,311 contigs that were longer than 2500 nucleotides, and described 0.70 and 0.79 million genes occurring in 179 and 248 genomes, as estimated by the mode of the frequency of bacterial single-copy core genes (Additional file 3). On average, 80.8% of the reads in donor metagenomes mapped back to the assembled contigs from donor metagenomes, which suggests that the assemblies represented a large fraction of the donor microbial communities. Donor assemblies recruited only 43.4% of the reads on average from the pre-FMT recipient metagenomes. This number increased to 80.2% for post-FMT recipient metagenomes and remained at an average of 76.8% even 1 year post-FMT (Additional file 3). These results suggest that members of the donor microbiota successfully established in the recipient gut and persisted long term.

To investigate functional determinants of microbial colonization by identifying donor populations that were successful at colonizing multiple individuals, we reconstructed microbial genomes from donor assemblies using sequence composition and differential coverage signal as previously described [34, 35]. We manually refined metagenomic bins to improve their quality following previously described approaches [36, 37] and only retained those that were at least 70% complete and had no more than 10% redundancy as predicted by bacterial single-copy core genes [38, 39]. Our binning effort resulted in a final list of 128 metagenome-assembled genomes (MAGs) for donor A and 183 MAGs for donor B that included members of Firmicutes (*n* = 265), Bacteroidetes (*n* = 20), Actinobacteria (*n* = 14), Proteobacteria (*n* = 7), Verrucomicrobia (*n* = 2), Cyanobacteria (*n* = 2), and Patescibacteria (*n* = 1) (Additional file 4). The taxonomy of donor-derived genomes largely reflected the taxonomic composition of donor metagenomic short reads (Fig. 1, Additional file 3, Additional file 4). While only 20 genomes (mostly of the genera *Bacteroides* and *Alistipes*) explained the entirety of the Bacteroidetes group, we recovered 265 genomes that represented lower abundance but diverse populations of Firmicutes (Fig. 1, Additional file 3, Additional file 4).

### Metagenomic read recruitment elucidates colonization events

Reconstructing donor genomes enabled us to characterize (1) population-level microbial colonization dynamics before and after FMT using donor and recipient metagenomes and (2) the distribution of each donor population across geographically distributed humans using 1984 publicly available human gut metagenomes (Fig. 1, Additional file 5).

Our metagenomic read recruitment analysis showed that donor A and B genomes recruited on average 77.05 and 83.04%, respectively, of reads from post-FMT metagenomes, suggesting that the collection of donor genomes well represents the recipient metagenomes post-FMT (Fig. 1). As expected, we detected each donor population in at least one donor metagenome (see “Methods” for “detection” criteria). Yet, only 16% of donor A populations were detected in every donor A sample, and only 44% of donor B populations were detected in every donor B sample (Fig. 1, Additional file 4), demonstrating the previously documented dynamism of gut microbial community composition over time [11]. A marked increase in the detection of donor populations in recipients after FMT is in agreement with the general pattern of transfer suggested by the short-read taxonomy (Fig. 1): while we detected only 38% of donor A and 54% of donor B populations in at least one recipient pre-FMT, these percentages increased to 96% for both donors post-FMT (Additional file 4). We note that we observed a higher fraction of donor populations in recipients as a function of the FMT delivery method. Following the cases of FMT where donor stool was transplanted via colonoscopy, we detected 54.7 and 33.3% donor genomes in the recipients of donor A (*n* = 3) and donor B (*n* = 2), respectively. In contrast, in the cases of FMT where donor stool was transplanted via pills, we detected 69.5 and 61.6% donor genomes in the recipients of donor A (*n* = 2) and donor B (*n* = 3), respectively.

Overall, not every donor population in our dataset was detected in each recipient, but the emergence of donor populations in recipients did not appear to be random: while some donor populations colonized all recipients, others colonized none (Fig. 1), providing us with an opportunity to quantify colonization success for each donor population in our dataset.

### Succession of donor microbial populations in FMT recipients and their prevalence in publicly available metagenomes reveal good and poor colonizers

Of the populations that consistently occurred in donor metagenomes, some were absent in all or most recipient metagenomes after FMT, and others were continuously present throughout the sampling period in both donor and recipient metagenomes (Fig. 1). To gain insights into the ecology of donor microbial populations beyond our dataset, we explored their occurrence in publicly available healthy gut metagenomes through metagenomic read recruitment. This analysis enabled us to consider the prevalence of donor populations in FMT recipients and global gut metagenomes, and define two groups of donor genomes that represented opposite colonization and prevalence phenotypes.

The “good colonizers” comprise those microbial populations that colonized and persisted in all FMT recipients. Intriguingly, these populations were also the most prevalent in publicly available gut metagenomes from Canada. Overall, these donor microbial populations (1) systematically colonized the majority of FMT recipients, (2) persisted in these environments long-term regardless of host genetics or lifestyle, and (3) were prevalent in public gut metagenomes outside of our study. In contrast, the so-called “poor colonizers” failed to colonize or persist in at least three FMT recipients. These populations were nevertheless viable in the donor gut environment: not only did they occur systematically in donor metagenomes but also they sporadically colonized some FMT recipients. Yet, unlike the good colonizers, the distribution patterns of poor colonizers were sparse within our cohort, as well as within the publicly available metagenomes. In fact, populations identified as poor colonizers were less prevalent than good colonizers in each of the 17 different countries we queried. In countries including the USA, Canada, Austria, China, England, and Australia, microbial populations identified as good colonizers occurred in 5 times more people than poor colonizers in the same country (Fig. 1, Additional file 4), which suggests that the outcomes of FMT in our dataset were unlikely determined by neutral processes. This observation is in contrast with previous studies that suggested “dose” (i.e., the abundance of a given population in donor fecal matter) as a predominant force that determines outcomes of colonization after FMT [40, 41]. However, our strain-resolved analysis of colonization events in our data in conjunction with the distribution of the same populations in publicly available metagenomes (1) revealed a significant correlation between the colonization success of donor populations and their prevalence across publicly available metagenomes, and (2) showed that the prevalence of a given population across global gut metagenomes can predict its colonization success after FMT better than its abundance in the donor stool sample (Wald test, *p* = 6.3e − 06 and *p* = 9.0e − 07) (Additional file 6). Overall, these observations suggest a link between the colonization outcomes in our study and global prevalence of the same microbial populations and that the succession of donor populations in our data were likely influenced by selective processes that influence colonization outcomes.

**Fig. 2:**
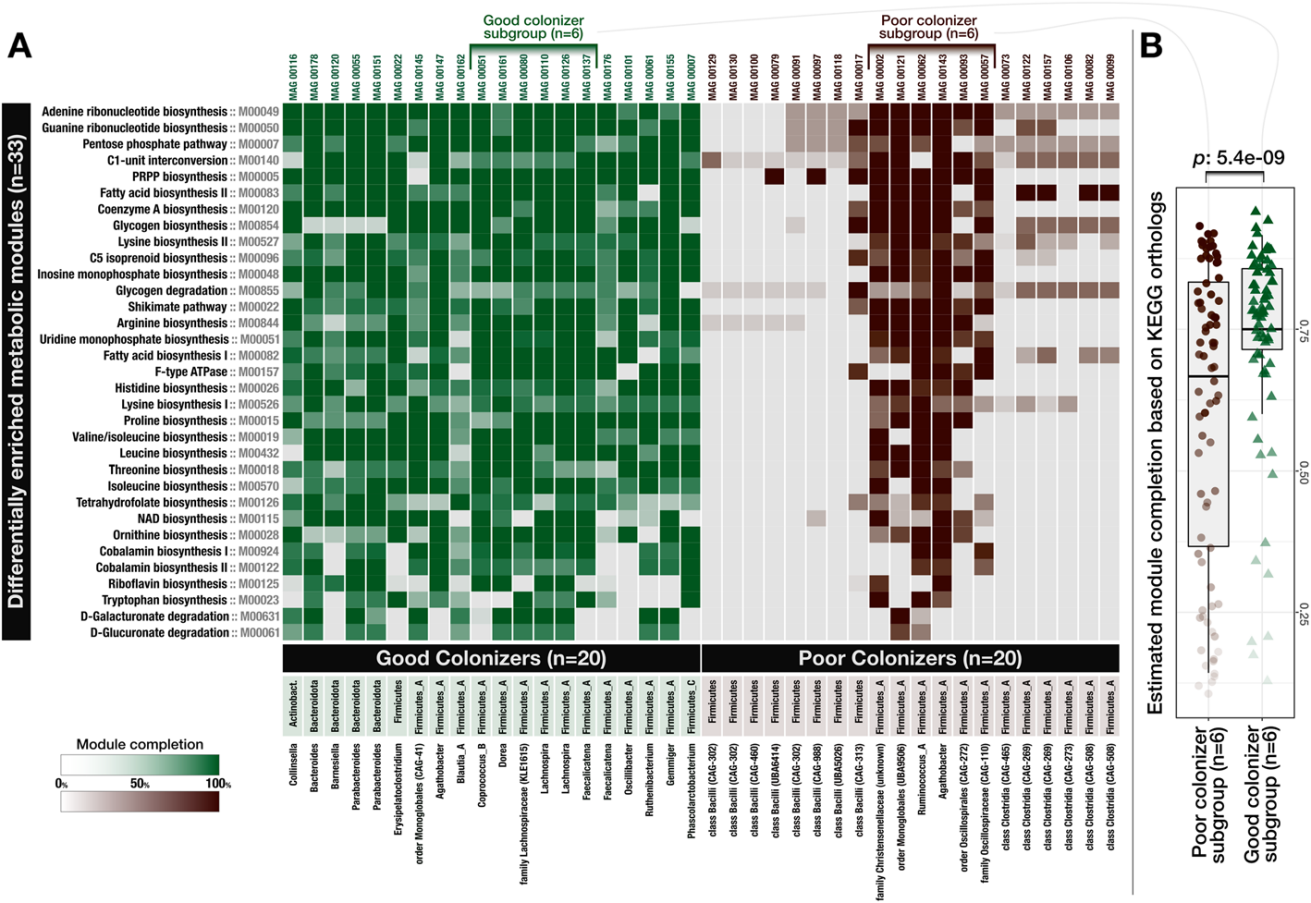
Distribution of metabolic modules across genomes of good and poor colonizers. Each data point in this heat map **A** shows the level of completion of a given metabolic module (rows) in a given genome (columns). The box-plot on the right-side **B** compares a subset of poor colonizer and good colonizer genomes, where each data point represents the level of completion of a given metabolic module in a genome and shows a statistically significant difference between the overall completion of metabolic modules between these subgroups (Wilcoxon rank sum test, *p* = 5.4e − 09). A high-resolution version of this figure is also available at https://doi.org/10.6084/m9.figshare.15138720 [26]

Next, we sought to investigate whether we can identify metabolic features that systematically differ between good colonizers and poor colonizers independent of their taxonomy. To conduct such a comparative analysis, we conservatively selected the top 20 populations from each group that best reflect their group properties by considering both their success after FMT and their prevalence across publicly available metagenomes (Additional file 9). The 20 populations representative of good colonizers were dominated by Firmicutes (15 of 20) but also included Bacteroidetes and one Actinobacteria population. All populations identified as poor colonizers resolved to Firmicutes (Fig. 2, Additional file 9). Genome completion estimates did not differ between good and poor colonizers (Wilcoxon rank sum test, *p* = 0.42) and averaged to 91 and 93%, respectively. But intriguingly, the genome sizes between the two groups differed dramatically (*p* = 2.9e − 06): genomes of good colonizers averaged to 2.8 Mbp while those of poor colonizers averaged to 1.6 Mbp. We considered that our bioinformatics analyses may have introduced biases to genome lengths, but found a very high correspondence between the lengths of the genomes and their best matching reference genomes in the Genome Taxonomy Database (GTDB) (R^2^ = 0.88, *p* = 5e − 14). Assuming that the generally larger genomes of good colonizers may be an indication of an increased repertoire of core metabolic competencies compared to poor colonizers, we next conducted a metabolic enrichment analysis for quantitative insights (see “Methods”).

### Good colonizers are enriched in metabolic pathways for the biosynthesis of essential organic compounds

Our enrichment analysis between good and poor colonizers revealed 33 metabolic modules (out of 443 total in the KEGG module database) that were enriched in good colonizers and none that were enriched in poor colonizers (Fig. 2, Additional file 9). Of all enriched modules, 79% were related to biosynthesis, indicating an overrepresentation of biosynthetic capabilities among good colonizers as KEGG modules for biosynthesis only make up 55% of all KEGG modules (Fig. 2, Additional file 9). Of the 33 enriched modules, 48.5% were associated with amino acid metabolism, 21.2% with vitamin and cofactor metabolism, 18.2% with carbohydrate metabolism, 24.2% with nucleotide metabolism, 6% with lipid metabolism, and 3% with energy metabolism (Additional file 9). Metabolic modules that were enriched in the good colonizers included the biosynthesis of seven of nine essential amino acids, indicating the importance of high metabolic independence to synthesize essential compounds as a likely factor that increases success in colonizing new environments (Additional file 9). This is further supported by the enrichment of biosynthesis pathways for the essential cofactor vitamin B12 (cobalamin), which occurred in 67.5% of the good colonizers and only 12.5% of the poor colonizers (Additional file 9). Vitamin B12 is structurally highly complex and costly to produce, requiring expression of more than 30 genes that are exclusively encoded by bacteria and archaea [42]. In addition to the biosynthesis of tetrahydrofolate, riboflavin, and cobalamin, the genomes of good colonizers had a larger representation of biosynthetic modules for vitamins including biotin, pantothenate, folate, and thiamine (Additional file 9). These micronutrients are equally essential in bacterial and human metabolism and are important mediators of host-microbe interactions [43]. Interestingly, enriched metabolic modules in our analysis partially overlap with those that Feng et al. identified as the determinants of microbial fitness using metatranscriptomics and a germ-free mouse model conventionalized with microbial isolates of human origin [7].

Even though these 33 metabolic modules were statistically enriched in populations identified as good colonizers, some of them also occurred in the genomes of poor colonizers (Fig. 2). To identify whether the levels of completion of these modules could distinguish the good and poor colonizers, we matched six good colonizers that encoded modules enriched in these populations to six populations of poor colonizers from the same phylum (Fig. 2). Bacterial single-copy core genes estimated that genomes in both subgroups were highly complete with a slight increase in average genome completion of poor colonizers (93.7%) compared to good colonizers (90.1%). Despite the higher estimated genome completion for populations of poor colonizers, estimated metabolic module completion values were slightly yet significantly lower in this group (Wilcoxon rank sum test with continuity correction, *V* = 958, *p* = 5e − 09) (Fig. 2, Additional file 9). Thus, these modules were systematically missing genes in populations of poor colonizers, indicating their functionality was likely reduced, if not absent.

These observations suggest that the ability to synthesize cellular building blocks, cofactors, and vitamins required for cellular maintenance and growth provides a substantial advantage during secondary succession, highlighting that the competitive advantages conferred by metabolic autonomy may outweigh the additional costs under certain conditions. For the remainder of our study, we use the term “high metabolic independence” (HMI) to describe genomic evidence for a population’s ability to synthesize essential compounds (that is, high completeness scores of biosynthesis pathways for these compounds indicating the presence of most, if not all, genes required to produce them), and “low metabolic independence” (LMI) to describe the absence of, or reduction in, such capacity.

### While gut microbial ecosystems of healthy individuals include microbes with both low- and high-metabolic independence, IBD primarily selects for microbes with high-metabolic independence.

Our results so far show that while the healthy donor environment could support both HMI and LMI populations (Fig. 1, Additional file 4), challenging microbes to colonize a new environment or to withstand ecosystem perturbation during FMT selects for HMI populations (Fig. 2, Additional file 9), suggesting that metabolic independence is a more critical determinant of fitness during stress than during homeostasis. Based on these observations, it is conceivable to hypothesize that (1) a gut environment in homeostasis will support a large variety of microbial populations with a wide spectrum of metabolic independence, and (2) a gut environment under stress will select for populations with high metabolic independence, potentially leading to an overall reduction in diversity.

To test these hypotheses, we compared genomes reconstructed from a cohort of healthy individuals [44] to genomes reconstructed from individuals who were diagnosed with inflammatory bowel disease (IBD). Our IBD dataset was composed of two cohorts: a set of patients with pouchitis [22], a form of IBD with similar pathology to ulcerative colitis [45], and a set of pediatric Crohn’s disease patients [46]. The number of genomes per individual and the average level of genome completeness per group were similar between healthy individuals and those with IBD: overall, our analysis compared 264 genomes from 22 healthy individuals with an average completion of 90.4%, 44 genomes from 4 pouchitis patients with an average completion of 89.2% and 256 genomes from 12 Crohn’s disease patients with an average completion of 94.1% (Additional file 10). Intriguingly, similar to the size differences between genomes of HMI populations and LMI populations (2.8 Mbp versus 1.6 Mbp on average), genomes of microbial populations associated with IBD patients were larger compared to those of microbial populations in healthy people and averaged to 3.0 Mbp versus 2.6 Mbp, respectively (Additional file 10). This suggests that the environmental filters created by FMT and gastrointestinal inflammation both select for microbial populations with larger genomes and potentially higher metabolic independence.

**Fig. 3:**
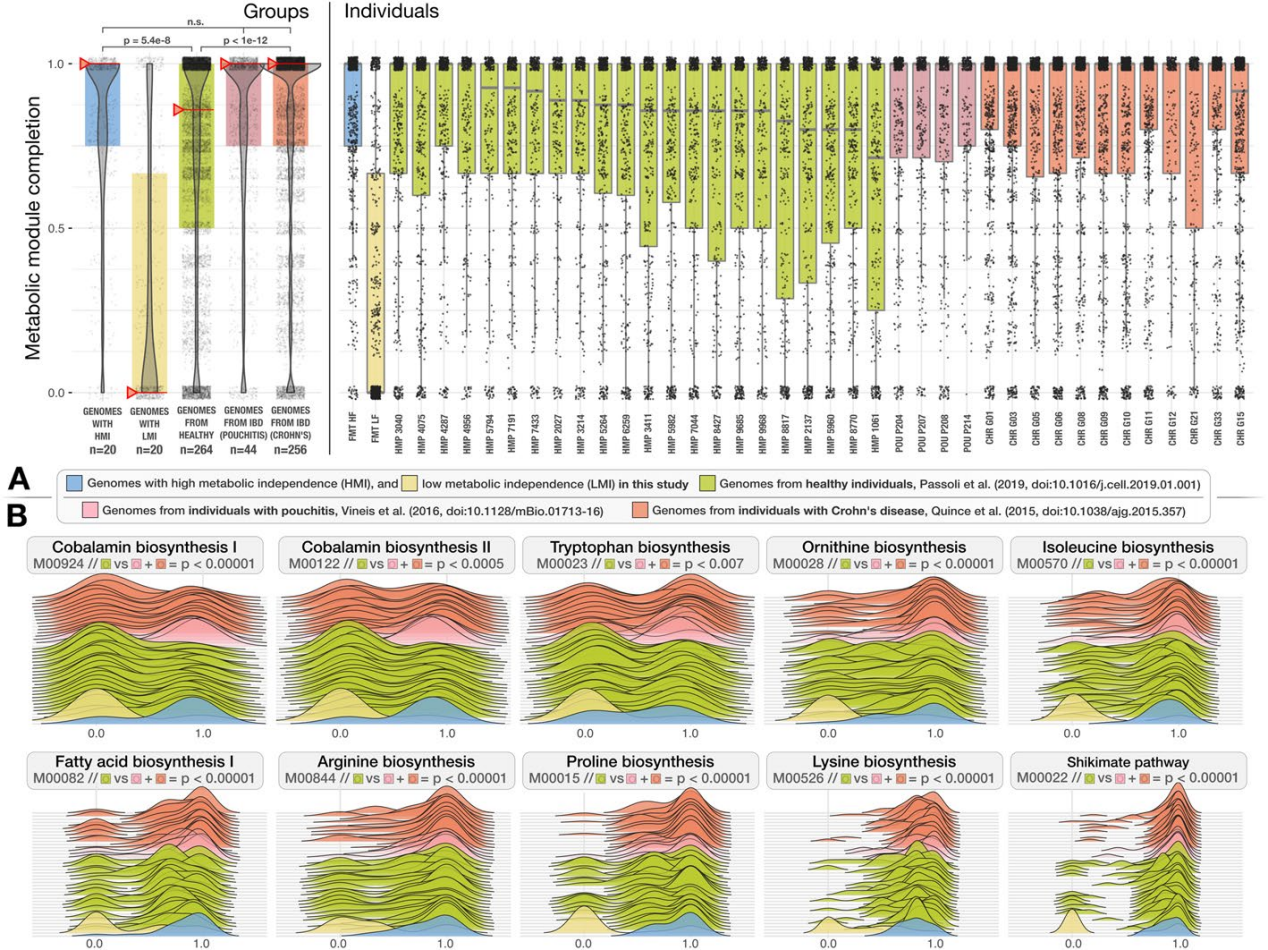
Distribution of metabolic modules in genomes reconstructed from healthy individuals and individuals with IBD. The boxplots in **A** show the metabolic module completion values for (1) high and (2) low metabolic independence donor genomes identified in this study (blue and yellow), (3) genomes from healthy individuals (green), and (4) genomes from individuals with pouchitis (red) and Crohn’s disease (orange). Each dot in a given box-plot represents one of 33 metabolic modules that were enriched in HMI FMT donor populations and the *y*-axis indicates its estimated completion. The leftmost panel in **A** represents group averages and red whiskers indicate the median. The rightmost panel in **A** shows the distribution of metabolic modules for individuals within each group. In **B** the completion values for 10 of the 33 pathways are demonstrated as ridge-line plots. Each plot represents a single metabolic module where each layer corresponds to an individual, and the shape of the layer represents the completion of a given metabolic module across all genomes reconstructed from that individual. A high-resolution version of this figure is also available at https://doi.org/10.6084/m9.figshare.15138720 [26]

Next, we asked whether the completion of metabolic modules associated with colonization success and resilience during FMT differed between the genomes reconstructed from healthy and IBD individuals. The completion of the 33 metabolic modules was almost identical between the HMI populations revealed by FMT and microbial populations in IBD patients (Wilcoxon rank sum test, *p* = 0.5) (Fig. 3, Additional file 10). In contrast, the completion of these metabolic modules was significantly reduced in microbial populations in healthy individuals (Wilcoxon rank sum test, *p* < 1e − 07) (Fig. 3, Additional file 10). Metabolic modules with the largest differences in completion between genomes from healthy and IBD individuals included biosynthesis of cobalamin, arginine, ornithine, tryptophan, isoleucine, and the Shikimate pathway (Fig. 3, Additional file 10), a seven-step metabolic route bacteria use for the biosynthesis of aromatic amino acids (phenylalanine, tyrosine, and tryptophan) [47].

Our findings show that the same set of biosynthetic metabolic modules that distinguish good and poor colonizers during FMT were also differentially associated with populations of IBD patients and healthy individuals. In particular, while healthy individuals harbored microbes with a broad spectrum of metabolic capacity, microbes from individuals who suffer from two different forms of IBD had significantly higher biosynthetic independence. It is conceivable that a stable gut microbial ecosystem is more likely to support LMI populations through metabolic cross-feeding, where vitamins, amino acids, and nucleotides are exchanged between microbes [48]. In contrast, host-mediated environmental stress in IBD likely disrupts such interactions and creates an ecological filter that selects for metabolic independence, which subsequently leads to loss of diversity and the dominance of organisms with large genomes that are often not as abundant or as competitive in states of homeostasis.

These observations have implications for our understanding of the hallmarks of healthy gut microbial ecosystems. Defining the “healthy gut microbiome” has been a major goal of human gut microbiome research [49], which still remains elusive [50]. Despite comprehensive investigations that considered core microbial taxa [51, 52] or guilds of microbes that represent coherent functional groups [53], the search for “biomarkers” of healthy gut microbiomes is ongoing [54]. Our findings indicate that beyond the taxonomic diversity of a microbial community, a broad range of metabolic independence represents a defining feature of a healthy gut microbiome. Conversely, our findings also suggest that an enrichment of metabolically independent populations could serve as an indicator of environmental stress in the human gut. Detection of these metabolic markers is not influenced by fluctuations in taxonomic composition or diversity, and represents a quantifiable feature of microbial communities through genome-resolved metagenomic surveys.

Our findings offer a new, taxonomy-independent perspective on the determinants of microbial resilience in the human gut environment under stress. Yet, our study is limited to well-known metabolic pathways—which, given the extent of the unknown coding space in microbial genomes [55], are likely far from complete—as well as by our ability to recognize gene function, which is determined by the sequences described in public databases that favor well-studied microbial organisms (Additional file 6). Thus, conservatively put, the enrichment of biosynthetic modules in HMI populations suggests that the ability to synthesize essential biological compounds is necessary but likely insufficient to survive environmental stress in the gut. Nevertheless, the finding that the same metabolic modules that promote colonization success after FMT are also the hallmarks of resilience in IBD suggests the presence of unifying ecological principles that govern microbial diversity in distinct modes of stress, which warrants deeper investigation.

## Conclusions

Our study identifies high metabolic independence conferred by the biosynthetic capacity for amino acids, nucleotides, and essential micronutrients as a distinguishing hallmark of microbial populations that colonize recipients of FMT and that thrive in IBD patients. These findings highlight the functional complexity of the human gut microbiome whose various interactions with the host are shaped through a network of microbial interactions such as cross-feeding of macro- and micronutrients. Our study offers a simple model that posits the following: microbial populations that are metabolically independent and those that lack the means to synthesize essential metabolites co-occur in a healthy gut environment in harmony, where their differential resilience to stress is indiscernible by their taxonomy or relative abundance. However, the challenges associated with the transfer to a new gut environment through FMT, or with host-mediated stress through IBD, initiate an ecological filter that selects for microbes that can self-sustain in the absence of ecosystem services associated with states of homeostasis. This model provides a hypothesis that explains the dominance of low-abundance members of healthy gut environments under stressful conditions, without any necessary direct causal association with disease state. If the association between particular microbial taxa and disease is solely driven by their superior metabolic independence, microbial therapies that aim to treat complex diseases by adding microbes associated with healthy individuals will be unlikely to compete with the adaptive processes that regulate complex gut microbial ecosystems.

## Methods

### Sample collection and storage

We selected our samples from a subset of individuals who participated in a randomized clinical trial [56]. Our selection criteria took into consideration multiple factors that were not applicable to all participants of the clinical study. Briefly, we aimed to identify (1) donors that contributed a large number of fecal samples over long periods of time (to maximize the number and quality of genomes from metagenomes and to be able to identify the extent of intrapersonal variability of the microbiota and its potential impact on our results), (2) donors whose feces were transplanted to the largest number of recipients (to be able to discuss the colonization dynamics of the same donor populations in different individuals accurately), (3) multiple recipients for each donor that received FMT via different methods, such as colonoscopy versus pills (to be able to better understand the generalizability of our downstream observations independent of the delivery method), and (4) recipients that were followed the longest period of time after FMT (to be able to follow donor population dynamics accurately). We did not consider factors that may impact the microbial community composition (such as age, gender, or diet) to homogenize the recipient cohort to observe overarching microbial patterns after FMT that are beyond environmental factors dictated by the host. Based on these criteria, we identified two donors (DA and DB), and 5 FMT recipients for each donor. All recipients received vancomycin for a minimum of 10 days pre-FMT at a dose of 125 mg four times daily. Three DA and two DB recipients received FMT via pill, and two DA and three DB recipients received FMT via colonoscopy. All recipients had recurrent *C. difficile* infection before FMT, and two DA recipients and one DB recipient were also diagnosed with ulcerative colitis (UC). Twenty-four stool samples were collected from the DA donor over a period of 636 days, and 15 stool samples were collected from the DB donor over a period of 532 days. Between 5 and 9 stool samples were collected from each recipient over periods of 187 to 404 days, with at least one sample collected pre-FMT and 4 samples collected post-FMT. This gave us a total of 109 stool samples from all donors and recipients. Samples were stored at − 80 °C (Additional file 2: Fig. S1, Additional file 1).

### Metagenomic short-read sequencing

We extracted the genomic DNA from frozen samples according to the centrifugation protocol outlined in MoBio PowerSoil kit with the following modifications: cell lysis was performed using a GenoGrinder to physically lyse the samples in the MoBio Bead Plates and Solution (5–10 min). After final precipitation, the DNA samples were resuspended in TE buffer and stored at − 20 °C until further analysis. Sample DNA concentrations were determined by PicoGreen assay. DNA was sheared to ~ 400 bp using the Covaris S2 acoustic platform, and libraries were constructed using the Nugen Ovation Ultralow kit. The products were visualized on an Agilent Tapestation 4200 and size-selected using BluePippin (Sage Biosciences). The final library pool was quantified with the Kapa Biosystems qPCR protocol and sequenced on the Illumina NextSeq500 in a 2 × 150 paired-end sequencing run using dedicated read indexing.

### ‘Omics workflows

Whenever applicable, we automated and scaled our ‘omics analyses using the bioinformatics workflows implemented by the program “anvi-run-workflow” [37] in anvi’o 7.1 [57, 58]. Anvi’o workflows implement numerous steps of bioinformatics tasks including short-read quality filtering, assembly, gene calling, functional annotation, hidden Markov model search, metagenomic read recruitment, metagenomic binning, and phylogenomics. Workflows use Snakemake [59] and a tutorial is available at the URL http://merenlab.org/anvio-workflows/ [60]. The following sections detail these steps.

### Taxonomic composition of metagenomes based on short reads

We used Kraken2 v2.0.8-beta [61] with the NCBI’s RefSeq bacterial, archaeal, viral, and viral neighbors genome databases to calculate the taxonomic composition within short-read metagenomes.

### Assembly of metagenomic short reads

To minimize the impact of random sequencing errors in our downstream analyses, we used the program “iu-filter-quality-minoche” to process short metagenomic reads, which is implemented in illumina-utils v2.11 [62] and removes low-quality reads according to the criteria outlined by Minoche et al. [63]. IDBA_UD v1.1.2 [64] assembled quality-filtered short reads into longer contiguous sequences (contigs), although we needed to recompile IDBA_UD with a modified header file so it could process 150 bp paired-end reads.

### Processing of contigs

We use the following strategies to process both sequences we obtained from our assemblies and those we obtained from reference genomes. Briefly, we used (1) “anvi-gen-contigs-database” on contigs to compute k-mer frequencies and identify open reading frames (ORFs) using Prodigal v2.6.3 [65], (2) “anvi-run-hmms” to identify sets of bacterial [66] and archaeal [67] single-copy core genes using HMMER v3.2.1 [68], (3) “anvi-run-ncbi-cogs” to annotate ORFs with functions from the NCBI’s Clusters of Orthologous Groups (COGs) [69], and (4) “anvi-run-kegg-kofams” to annotate ORFs with functions from the KOfam HMM database of KEGG orthologs (KOs) [70, 71]. To predict the approximate number of genomes in metagenomic assemblies, we used the program “anvi-display-contigs-stats,” which calculates the mode of the frequency of single-copy core genes as described previously [72].

### Metagenomic read recruitment, reconstructing genomes from metagenomes, determination of genome taxonomy, and ANI

We recruited metagenomic short reads to contigs using Bowtie2 v2.3.5 [73] and converted resulting SAM files to BAM files using samtools v1.9 [74]. We profiled the resulting BAM files using the program “anvi-profile” with the flag “–min-contig-length” set to 2500 to eliminate shorter sequences to minimize noise. We then used the program “anvi-merge” to combine all read recruitment profiles into a single anvi’o merged profile database for downstream visualization, binning, and statistical analyses (the https://doi.org/10.6084/m9.figshare.14331236 [75] gives access to reproducible data objects). We then used “anvi-cluster-contigs” to group contigs into 100 initial bins using CONCOCT v1.1.0 [76], “anvi-refine” to manually curate initial bins with conflation error based on tetranucleotide frequency and differential coverage signal across all samples, and “anvi-summarize” to report final summary statistics for each gene, contig, and bin. We used the program “anvi-rename-bins” to identify bins that were more than 70% complete and less than 10% redundant, and store them in a new collection as metagenome-assembled genomes (MAGs), discarding lower quality bins from downstream analyses. GTBD-tk v0.3.2 [77] assigned taxonomy to each of our MAGs using GTDB r89 [78], but to assign species- and subspecies-level taxonomy for “DA_MAG_00057,” “DA_MAG_00011,” “DA_MAG_00052,” and “DA_MAG_00018,” we used “anvi-get-sequences-for-hmm-hits” to recover DNA sequences for bacterial single-copy core genes that encode ribosomal proteins, and searched them in the NCBI’s nucleotide collection (nt) database using BLAST [79]. Finally, the program “anvi-compute-genome-similarity” calculated pairwise genomic average nucleotide identity (gANI) of our genomes using PyANI v0.2.9 [80].

### Criteria for MAG detection in metagenomes

Using mean coverage to assess the occurrence of populations in a given sample based on metagenomic read recruitment can yield misleading insights, since this strategy cannot accurately distinguish reference sequences that represent very low-abundance environmental populations from those sequences that do not represent an environmental population in a sample yet still recruit reads from non-target populations due to the presence of conserved genomic regions. Thus, we relied upon the “detection” metric, which is a measure of the proportion of the nucleotides in a given sequence that are covered by at least one short read. We considered a population to be detected in a metagenome if anvi’o reported a detection value of at least 0.25 for its genome (whether it was a metagenome-assembled or isolate genome). Values of detection in metagenomic read recruitment results often follow a bimodal distribution for populations that are present and absent (see Additional file 2: Fig. S2 in ref. [81]), thus 0.25 is an appropriate cutoff to eliminate false-positive signal in read recruitment results for populations that are absent.

### Identification of MAGs that represent multiple subpopulations

To identify subpopulations of MAGs in metagenomes, we used the anvi’o command “anvi-gen-variability-profile” with the “–quince-mode” flag which exported single-nucleotide variant (SNV) information for all MAGs after read recruitment. We then used DESMAN v2.1.1 [82] to analyze SNVs to determine the number and distribution of subpopulations represented by a single genome. To account for non-specific mapping that can inflate the number of estimated subpopulations, we removed any subpopulation that made up less than 1% of the entire population explained by a single MAG. To account for noise due to low coverage, we only investigated subpopulations for MAGs for which the mean non-outlier coverage of single-copy core genes was at least 10X.

### Criteria for colonization of a recipient by a MAG for colonization dynamics analyses (Additional file 6)

We applied the set of criteria described in Additional file 2: Fig. S4 to determine whether or not a MAG successfully colonized a recipient, and to confidently assign colonization or non-colonization phenotypes to each MAG/recipient pair where the MAG was detected in the donor sample used for transplant into the recipient. If these criteria were met, we then determined whether the MAG was detected in any post-FMT recipient sample taken more than 7 days after transplant. If not, the MAG/recipient pair was considered a non-colonization event. If the MAG was detected in the recipient greater than 7 days post-FMT, we used subpopulation information to determine if any subpopulation present in the donor and absent in the recipient pre-FMT was detected in the recipient more than 7 days post-FMT. If this was the case, we considered this to represent a colonization event. See Additional file 2: Fig. S4 for a complete outline of all possible cases.

### Phylogenomic tree construction

To concatenate and align amino acid sequences of 46 single-copy core [66] ribosomal proteins that were present in all of our *Bifidobacterium* MAGs and reference genomes, we ran the anvi’o command “anvi-get-sequences-for-hmm-hits” with the “–return-best-hit,” “–get-aa-sequence,” and “—concatenate” flags, and the “–align-with” flag set to “muscle” to use MUSCLE v3.8.1551 [83] for alignment. We then ran “anvi-gen-phylogenomic-tree” with default parameters to compute a phylogenomic tree using FastTree 2.1 [84].

### Analysis of metabolic modules and enrichment

We calculated the level of completeness for a given KEGG module [85, 86] in our genomes using the program “anvi-estimate-metabolism,” which leveraged previous annotation of genes with KEGG orthologs (KOs) (see the section “Processing of contigs”). Then, the program “anvi-compute-functional-enrichment” determined whether a given metabolic module was enriched in a group of genomes based on the output from the program “anvi-estimate-metabolism.” The URL https://anvio.org/m/anvi-estimate-metabolism [87] serves a tutorial for this program which details the modes of usage and output file formats. The statistical approach for enrichment analysis is defined elsewhere [37], but briefly it computes enrichment scores for functions (or metabolic modules) within groups by fitting a binomial generalized linear model (GLM) to the occurrence of each function or complete metabolic module in each group, and then computing a Rao test statistic, uncorrected *p*-values, and corrected *q*-values. We considered any function or metabolic module with a *q*-value less than 0.05 to be “enriched” in its associated group if it was also at least 75% complete and present in at least 50% of the group members.

### Determination of MAGs representing good and poor colonizers for metabolic enrichment analysis

We classified MAGs as good colonizers if, in all 5 recipients, they were detected in the donor sample used for transplantation as well as the recipient more than 7 days post-FMT. We classified MAGs as poor colonizers as those that, in at least 3 recipients, were detected in the donor sample used for FMT but were not detected in the recipient at least 7 days post-FMT. We reduced the number of good colonizer MAGs to be the same as the number of poor colonizer MAGs for metabolic enrichment analysis by selecting only those populations that were the most prevalent in the Canadian gut metagenomes.

### Classification of high metabolic independence

We developed a script to calculate the pathwise completeness of the 33 KEGG modules that were enriched in good colonizers in this study to determine whether a given genome resembles HMI or LMI populations. The URL https://anvio.org/m/anvi-script-estimate-metabolic-independence [88] serves more information.

### Ordination plots

We used the R vegan v2.4–2 package “metaMDS” function to perform nonmetric multidimensional scaling (NMDS) with Horn-Morisita dissimilarity distance to compare taxonomic composition between donor, recipient, and global metagenomes. We visualized ordination plots using R ggplot2.

## Supplementary Information

**Additional file 1.** Description of FMT study and stool samples collected. **a** Description of FMT donor stool samples and SRA accession numbers. **b** Description of FMT recipient samples and SRA accession numbers. **c** Description of transplantation events.

**Additional file 2: Supplementary figures S1, S2, S3 and S4. Fig. S1.** Timeline of stool samples collected from FMT study. Each circle represents a stool sample collected from either an FMT donor or FMT recipient. The thicker, red vertical line at day 0 represents the FMT event for each recipient. FMT method (pill or colonoscopy) and FMT recipient health and disease state (C. diff - chronic recurrent *Clostridium difficile* infection, UC - ulcerative colitis) are indicated on the right. **Fig. S2.** Nonmetric multidimensional scaling (NMDS) ordination of the taxonomic composition of donor, recipient, and Canadian gut metagenomes at the genus level based on Morisita-Horn dissimilarity. Samples from the same participant are joined by lines with the earliest time point labeled. CAN: Canadian gut metagenomes, DA: donor A, DB: donor B, POST: recipients post-FMT, PRE: recipients pre-FMT. **Fig. S3.** Nonmetric multidimensional scaling (NMDS) ordination of the taxonomic composition of the donor and recipient metagenomes at genus level based on Morisita-Horn dissimilarity. Samples from the same participant are joined by lines with the earliest time point labeled. DA_POST: donor A recipients post-FMT, DA_PRE: donor A recipients pre-FMT, DA: donor A, DB_POST: donor B recipients post-FMT, DB_PRE: donor B recipients pre-FMT, DB: donor B. **Fig. S4.** A flowchart outlining our method to assign successful colonization, failed colonization, or undetermined colonization phenotypes to donor-derived populations in the recipients of that donor’s stool.

**Additional file 3.** Description of FMT metagenomes and co-assemblies. **a** Metagenome SRA accession numbers and number of metagenomic short-reads sequenced and mapped to co-assemblies and MAGs. **b**) Phylum level taxonomic composition of metagenomes. c) Genus level taxonomic composition of metagenomes. d) Summary statistics for contigs from metagenome co-assemblies. 

**Additional file 4.** Description of MAGs. **a** Summary statistics and taxonomic assignments for MAGs. **b** and **c** Detection of Donor A and Donor B MAGs in FMT metagenomes, respectively. **d** and **e** Detection of Donor A and Donor B MAGs in global gut metagenomes, respectively. **f** and **g** Detection summary statistics of Donor A and Donor B MAGs in global gut metagenomes, respectively. **h** and **i** Mean non-outlier coverage of Donor A and Donor B MAG single-copy core genes in FMT metagenomes.

**Additional file 5.** Accession numbers of gut metagenomes from 17 countries.

**Additional file 6.** Additional discussion and supplementary figures for **A** adaptive ecological forces are the primary drivers of microbial colonization; **B** considerations of annotation bias that reduce the number of genes with functional annotations for microbial populations that typically occur in healthy individuals.

**Additional file 7.** MAG subpopulation information. **a** and **b** Number of Donor A and Donor B MAG subpopulations detected in FMT metagenomes, respectively. **c** and **d** Subpopulation composition of Donor A and Donor B MAGs in FMT metagenomes, respectively.

**Additional file 8.** MAG/recipient pair colonization outcomes and MAG mean coverage in the 2nd and 3rd quartiles in stool samples used for transplantation.

**Additional file 9.** Description of HMI vs. LMI populations. **a** Taxonomic assignments and genome size estimates for high- and low-metabolic independence populations. **b** KEGG module completeness information for high- and low-metabolic independence populations. **c** Raw KEGG module enrichment information for high- and low-metabolic independence populations. **d** KEGG module enrichment and categorical information for the 33 modules enriched in high-metabolic independence populations. **e** and **f** Completeness information for the 33 modules enriched in high-metabolic independence populations in all high- and low-metabolic independence populations.

**Additional file 10.** **a** List of genomes from healthy individuals and individuals with IBD. **b** Module completion values across genomes.

**Additional file 11.** Review history.

## Data Availability

Raw sequencing data for donor and recipient metagenomes are stored under the NCBI BioProject PRJNA701961 (see Additional file 1 for accession numbers for each sample) [89]. The geographically distributed human gut metagenomes were obtained from previously published datasets (Additional file 5) [44, 90–104]. The URL https://merenlab.org/data/fmt-gut-colonization [105] serves a reproducible bioinformatics workflow and gives access to ad hoc scripts, usage instructions, and intermediate data objects to reproduce findings in our study. All ad hoc scripts also available under CC-BY 4.0 International license on Figshare (https://doi.org/10.6084/m9.figshare.22352989) [106].
