# Supplementary material for: Metabolic independence drives gut microbial colonization and resilience in health and disease
Source: Genome Biol. 2023 Apr 17;24:78. doi: 10.1186/s13059-023-02924-x (PMC10108530; doi:10.1186/s13059-023-02924-x)
Supplement: Supplementary file 2 — Additional file 2: Supplementary figures S1, S2, S3 and S4. Fig. S1. Timeline of stool samples collected from FMT study. Each circle represents a stool sample collected from either an FMT donor or FMT recipient. The thicker, red vertical line at day 0 represents the FMT event for each recipient. FMT method (pill or colonoscopy) and FMT recipient health and disease state (C. diff - chronic recurrent Clostridium difficile infection, UC - ulcerative colitis) are indicated on the right. Fig. S2. Nonmetric multidimensional scaling (NMDS) ordination of the taxonomic composition of donor, recipient, and Canadian gut metagenomes at the genus level based on Morisita-Horn dissimilarity. Samples from the same participant are joined by lines with the earliest time point labeled. CAN: Canadian gut metagenomes, DA: donor A, DB: donor B, POST: recipients post-FMT, PRE: recipients pre-FMT. Fig. S3. Nonmetric multidimensional scaling (NMDS) ordination of the taxonomic composition of the donor and recipient metagenomes at genus level based on Morisita-Horn dissimilarity. Samples from the same participant are joined by lines with the earliest time point labeled. DA_POST: donor A recipients post-FMT, DA_PRE: donor A recipients pre-FMT, DA: donor A, DB_POST: donor B recipients post-FMT, DB_PRE: donor B recipients pre-FMT, DB: donor B. Fig. S4. A flowchart outlining our method to assign successful colonization, failed colonization, or undetermined colonization phenotypes to donor-derived populations in the recipients of that donor’s stool. [file 13059_2023_2924_MOESM2_ESM.pdf]

Supplementary Figures

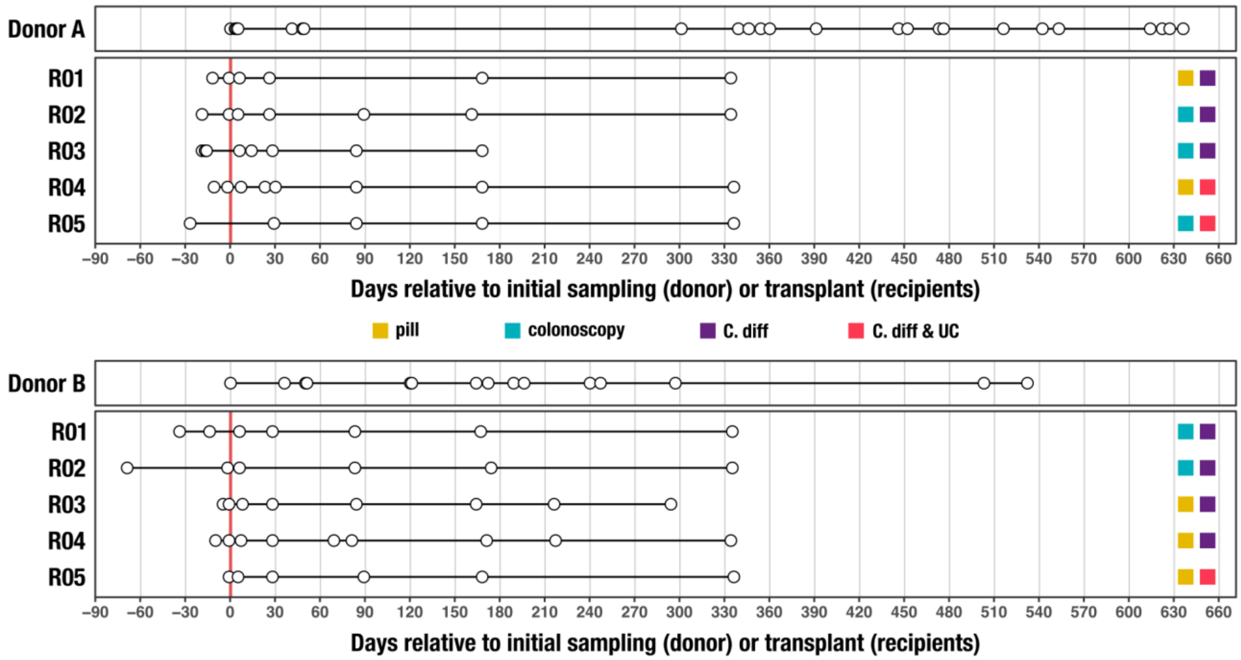

Fig. S1. Timeline of stool samples collected from FMT study. Each circle represents a stool sample collected from either an FMT donor or FMT recipient. The thicker, red vertical line at day 0 represents the FMT event for each recipient. FMT method (pill or colonoscopy) and FMT recipient health and disease state (C. diff - chronic recurrent *Clostridium difficile* infection, UC - ulcerative colitis) are indicated on the right.

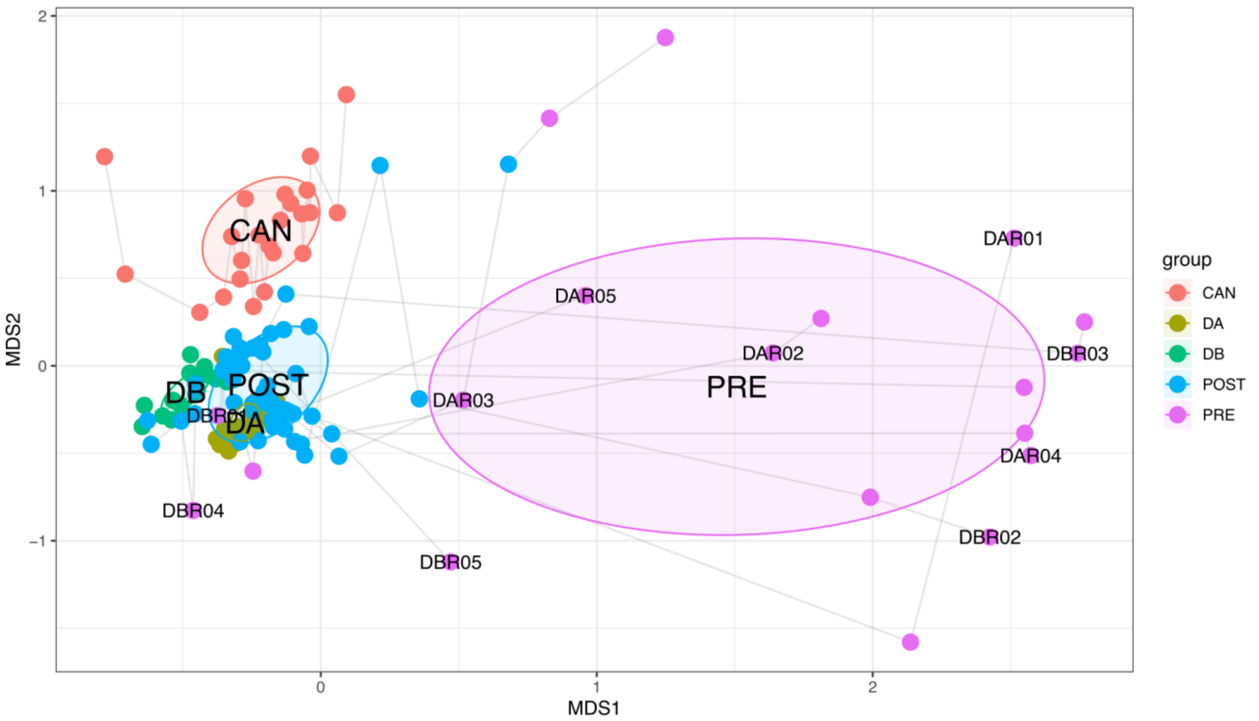

Fig. S2. Nonmetric multidimensional scaling (NMDS) ordination of the taxonomic composition of donor, recipient, and Canadian gut metagenomes at the genus level based on Morisita-Horn dissimilarity. Samples from the same participant are joined by lines with the earliest time point labeled. CAN: Canadian gut metagenomes, DA: donor A, DB: donor B, POST: recipients post-FMT, PRE: recipients pre-FMT.

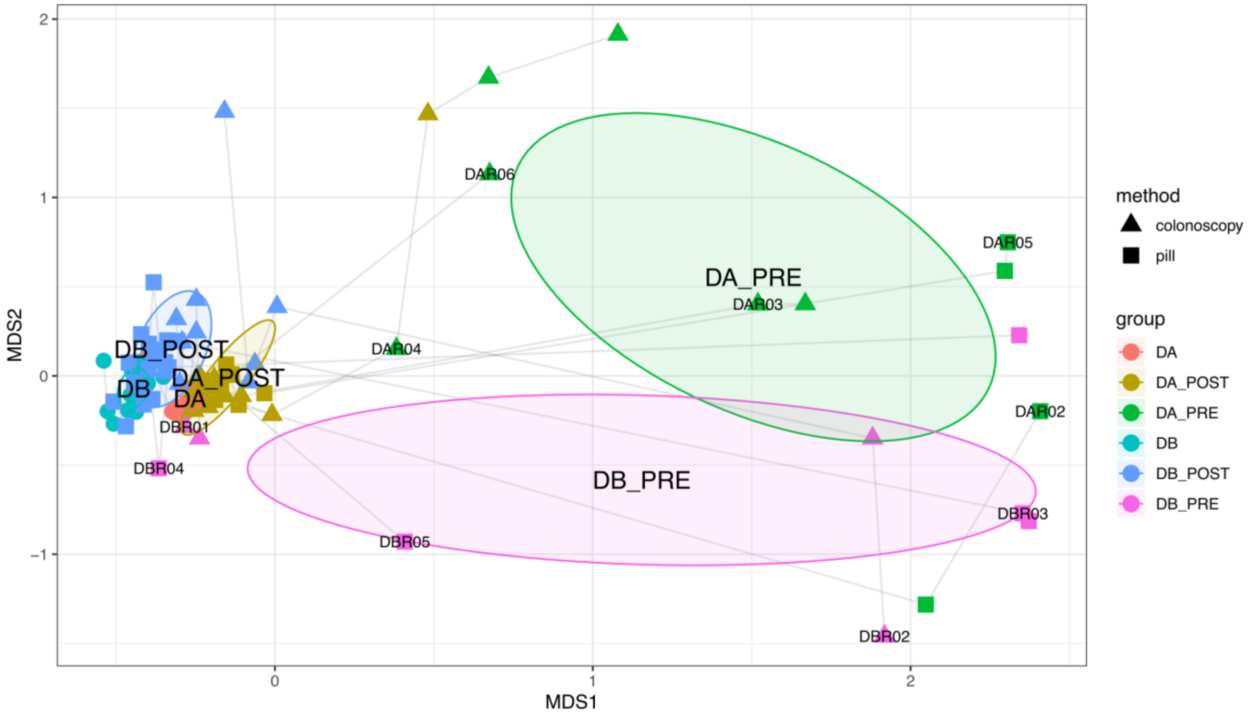

Fig. S3. Nonmetric multidimensional scaling (NMDS) ordination of the taxonomic composition of the donor and recipient metagenomes at genus level based on Morisita-Horn dissimilarity. Samples from the same participant are joined by lines with the earliest time point labeled. DA_POST: donor A recipients post-FMT, DA_PRE: donor A recipients pre-FMT, DA: donor A, DB_POST: donor B recipients post-FMT, DB_PRE: donor B recipients pre-FMT, DB: donor B.

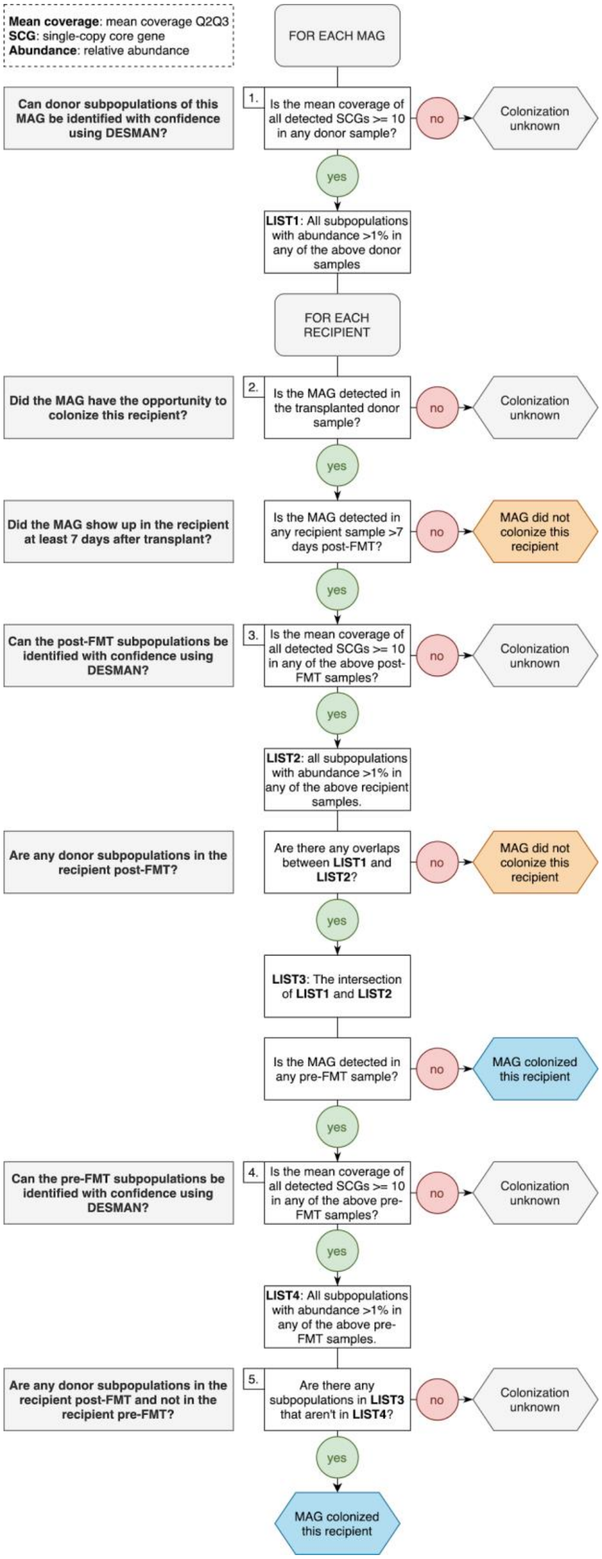

Fig. S4. A flowchart outlining our method to assign successful colonization, failed colonization, or undetermined colonization phenotypes to donor-derived populations in the recipients of that donor's stool.
