# Supplementary material for: Metabolic independence drives gut microbial colonization and resilience in health and disease
Source: Genome Biol. 2023 Apr 17;24:78. doi: 10.1186/s13059-023-02924-x (PMC10108530; doi:10.1186/s13059-023-02924-x)
Supplement: Supplementary file 11 — Additional file 11. Review history. [file 13059_2023_2924_MOESM11_ESM.docx]

Review History

**First round of review**

**Reviewer 1**

**Were you able to assess all statistics in the manuscript, including the appropriateness of statistical tests used?**

Yes, and I have assessed the statistics in my report.

**Comments to author:**

The manuscript by Watson, Füssel, Veseli et al. addresses an important question: what are the determinants of microbial colonization and succession. They examine 109 metagenomes from a previously published study of fecal transplants for C. difficile infection. They also compare this data to additional public metagenomes originating from differing countries as well as public IBD metagenomes. In my opinion the main novelty of this manuscript traces to their use of metagenomic assembly and binning across samples to characterize the genomic traits of successful colonizers. The authors also have made great efforts to make the data and resources available which should be commended.

I have 3 main concerns in the manuscript which can likely be addressed in revision:

1- The organisms/MAGs which can be successfully assembled will be the most abundant organisms in the community such as many of the Bacteroides spp. Identified by the authors. I would think that this creates a bias in which the ability to detect the organism in both recipients and the public metagenomes is skewed in the favour of these MAGs creating a false signal that the poor colonizers are less prevalent. Could the authors use their Kraken analysis to disprove this hypothesis and/or directly look at the dose hypothesis.

2- I am concerned that the observation that there is reduced functional/metabolic richness in poor colonizers may confounded by annotation bias. Most of the good colonizers have been matched to a well-known genus level while most of the poor colonizers can only be assigned to a class (Fig 2). These Bacilli and Clostridia also have very low module completion. As a function of the divergence from any well-characterized genome, I suspect that the functional annotations have been poorly propagated to these genomes.

3- The visualizations are extremely data dense to the point that they are borderline noneffective. I am still not sure I understand every track/panel of Figure 1. Because of the visualization choices it is often not possible to know what the units are or what the column/rows represent and/or spot the points being made by the authors in text. In the ridge plots of Figure 3, if I correctly understand what has been plotted, it is difficult to think that these would be statistically significant as indicated.

**Reviewer 2**

**Were you able to assess all statistics in the manuscript, including the appropriateness of statistical tests used?**

Yes, and I have assessed the statistics in my report.

**Comments to author:**

In this study, Andrea Watson et al investigated the association between metabolic modules of metagenomics and resilience in stressed gut environments, using fecal microbiota transplantation (FMT) experiment (2 donors and 10 recipients). The topic is of broad interest in this field. However, there are many major flaws in the study design, data analysis, and result interpretation. The overall conclusion is not supported by the current data (over concluded), and a more nuanced conclusion might be needed.

Specific comments:

- One of the major concerns is the small sample size (only 2 donors and 10 recipients). Reproducibility needs to be considered before making any meaningful conclusion. It is not clear why the authors only included a subset of individuals who participated in the randomized clinical trial.

- Importantly, the host factors (eg. age, sex, race, etc.) and the dietary factors, which could greatly affect the gut microbiome composition, were not considered at all in this study.

- Another concern is the determination of MAGs representing "good" and "poor" colonizers for metabolic enrichment analysis. The authors classified MAGs as "poor colonizers" as those that, 'in at least 3 recipients", were detected in the donor sample used for FMT but were not detected in the recipient at least 7 days post-FMT. Due to the small sample size of the study, this determination method (i.e. "in at least 3 recipients",) could be problematic.

- Page 11, Line 265. "enrichment analysis between good and poor colonizers revealed 33 metabolic modules that were enriched in good colonizers". How many metabolic modules were included in the analyses in total? This information is important for enrichment test, which needs to be described in the main text.

- Since this study has many analyses, thus multiple testing correction is needed.

- The author defined 'high metabolic independence' (HMI) as genomic evidence for a population's ability to synthesize essential compounds. Some more detailed criteria are needed for this definition.

- Figure 1. For each of the metagenome-assembled genomes, the species names need to be labeled. The current label like "00052" do not provide any meaningful information. In addition, it would be helpful to also highlight some of the known predominant bacteria taxa, such as Provotella and Bacteroides. What's the coverage for each of the metagenome-assembled genome? This kind of key information needs to be shown in Figure 1.

- It would be interesting to explore why 90% of the metagenome-assembled genomes in Figure 1 belonged to "Firmcutes" phylum. What happened to the other known predominant phylum, such as "Bacteroidetes" and "Proteobacteria"?

- Supplementary Figure 2 and 3. The beta-diversity analyses were performed on genus level. This resolution is not good enough, especially for metagenomics sequencing. Strain level analyses is needed, since this study have already assembled microbial genomes. In addition, I would suggest to use the phylogenetic based matrix, such as weighted UniFrac distance.

**Authors Response**

**Point-by-point responses to the reviewers’ comments:**

Reviewer #1:

The manuscript by Watson, Füssel, Veseli et al. addresses an important question: what are the determinants of microbial colonization and succession. They examine 109 metagenomes from a previously published study of fecal transplants for C. difficile infection. They also compare this data to additional public metagenomes originating from differing countries as well as public IBD metagenomes. In my opinion the main novelty of this manuscript traces to their use of metagenomic assembly and binning across samples to characterize the genomic traits of successful colonizers. The authors also have made great efforts to make the data and resources available which should be commended.

*Response: We thank the reviewer for their careful reading of our work and the time they have invested into evaluating it.*

I have 3 main concerns in the manuscript which can likely be addressed in revision:

The organisms/MAGs which can be successfully assembled will be the most abundant organisms in the community such as many of the Bacteroides spp. Identified by the authors. I would think that this creates a bias in which the ability to detect the organism in both recipients and the public metagenomes is skewed in the favour of these MAGs creating a false signal that the poor colonizers are less prevalent. Could the authors use their Kraken analysis to disprove this hypothesis and/or directly look at the dose hypothesis.

*Response: The reviewer correctly points out that genome-resolved surveys of metagenomes can be biased against low-abundance microbial populations, and in our case such biases could skew detection statistics to resolve the association between colonization ability and prevalence. Our recognition of this risk compelled us to generate not only very deeply sequenced metagenomes from our donors that were collected from at least 15 time points, but also focus on a set of samples that covered a period that spans across at least 532 days. We sequenced the donor metagenomes with the aim to generate over 65 million reads on average for each one of them, which resulted in 0.9 to 1.6 billion reads to characterize donor microbial population genomes alone. This strategy gave us a considerable advantage in two aspects of our genomic analyses that are lacking in most contemporary genome-resolved metagenomics surveys of the human gut: non-composite genome bins, and high rate of recovery for rare populations. The deep sequencing of our donor metagenomes, as well as the long period of sampling, dramatically improved our ability to assemble a large number of contigs and benefit from the differential coverage signal during the generation and refinement of metagenome-assembled genomes (MAGs). Secondly, by pooling reads from donors, we also were able to reconstruct genomes from rare populations, even if they were rare in every sample. In fact, 20% of the MAGs we have reconstructed had a maximum coverage of less than 5X across any of the donor samples despite the high sequencing depth, and 53% of them had a median coverage of less than 1X. These metrics indicate that the vast majority of MAGs were coming from low-abundance populations. For instance, DA_MAG_00090, a MAG that belongs to the phylum Firmicutes and resolves to the genus Neobitarella, offers a striking example for the extent of rarity we were able to characterize: the maximum detection value for DA_MAG_00090 in any of the donor metagenomes was only 0.6 – which means that its abundance was so low throughout the sampling period, only 60% of all nucleotides in this genome was covered by at least one short read during metagenomic read recruitment even in the metagenome it was most abundant, and its assembly was only possible by pooling reads from multiple timepoints. The median coverage of DA_MAG_00090 in the same set of metagenomes was only 0.23X, with a maximum coverage of 1.1X across the sampling period. But even for a population that almost never exceeds an average coverage of 1X, the detection statistic serves as a very good indicator of its presence in a metagenome. For instance, even for this extremely rare organism, our detection cutoff of 0.25 enabled us to characterize its distribution across donor and recipient metagenomes, which were even more deeply sequenced (an average of over 74 million reads per sample). While such statistics are buried in our Supplementary Tables (primarily S2 and S3), such low-abundance populations in fact represent the majority of our MAG pool, yet some of them showed much higher levels of detection in global metagenomes compared to our donors or recipients. While we do not exclude the fact that depth of sequencing will impact the detection of populations, we hope that these numbers offer a convincing argument that detection bias as a function of depth of sequencing should not be a significant concern for our study.*

*The reviewer proposes that our analyses of metagenomes with Kraken could help address the potential issues with detection bias. We respectfully disagree with this suggestion. Algorithms that offer taxonomic classification of short reads based on reference genomes suffer from significant technical challenges, including (1) the limited set of reference genomes poorly reflects the diversity of natural habitats, resulting in skewed representation of taxonomy especially at higher levels of resolution (such as below genus level), and (2) conserved regions of genomes that can be shared across relatively distantly related taxa typically inflates the observed number of low abundance populations with false negatives. In contrast, once a representative genomic catalog is recovered, the ability to quantify the ‘presence’ of similar populations in additional metagenomes becomes a much more powerful and reliable approach to quantify microbial population dynamics to assess phenomena such as dose dependence.*

– I am concerned that the observation that there is reduced functional/metabolic richness in poor colonizers may confounded by annotation bias. Most of the good colonizers have been matched to a well-known genus level while most of the poor colonizers can only be assigned to a class (Fig 2). These Bacilli and Clostridia also have very low module completion. As a function of the divergence from any well-characterized genome, I suspect that the functional annotations have been poorly propagated to these genomes.

*Response: The reviewer makes an excellent observation. And we agree with them that the annotation bias is something worth considering. Inspired by the point we conducted some additional analyses we share below. Although, before going into the details of this discussion we believe it is important to note that we also found a considerable size difference between good and poor colonizers. In fact, the good colonizers were over 1 Mbp larger than the poor colonizers on average (p=2.9e-06). This was the case even though the estimated completion of genomes based on single-copy core genes were similar between the two groups, with a slight increase in completion estimates for poor colonizers (91% and 93%, respectively). We believe the significant change in average genome sizes between good colonizers and poor colonizers already suggests that the reduced functional / metabolic richness in poor colonizers should not come across as surprising given the ~1,000 genes they lack.*

*To gain deeper insights into the annotation bias, we quantified the proportion of genes with functional annotation in publicly-available gut metagenomes. Our analysis of metagenomes that contained over 25 million reads showed that samples from healthy people indeed contained fewer annotations per gene call than metagenomes from people with inflammatory bowel disease, which is demonstrated in the figure below. This observation was not dependent on the annotation strategy, as we observed the same phenomenon both with a homology-based annotation method and a probabilistic one that used HMMs. We believe one conceivable interpretation of this result is that microbial populations that thrive in healthy individuals are less well-characterized than those that thrive in people with IBD. This has intriguing parallels to observations in our work.*

*It is indeed likely that those organisms that we have been able to cultivate are also those that are able to survive stressful conditions such as IBD; thus, one of the hallmarks of IBD is the dominating presence of well-characterized microbes. In contrast, healthy gut environments promote a larger representation of microbes that are difficult to isolate due to various factors, perhaps including their reduced genomes, and their absence in public databases therefore hampers annotation. It is definitely intriguing.*

*We believe a large fraction of reduced metabolic capacity we observe is due to biology (i.e., due to the systematic and significant reduction in genome sizes of poor colonizers), although we also agree with the reviewer that the limitations of functional annotation databases and the novelty of most of the poor colonizers have a non-zero contribution to the annotation bias we observe. Nevertheless, given that we use state-of-the-art approaches for functional annotation and metabolic reconstruction, and have no control over the progress in functional databases, we believe this intriguing discussion falls outside of the scope of our work. We are thankful to the reviewer for bringing up this point.*

*We are posting the visualization supporting this point to an anonymous image hosting service:*

*The histograms in this image shows annotations per gene call for deeply-sequenced metagenomes (>25 million reads) from a) Pfam, b) NCBI COGs, and c) KEGG KOfams. d) Histogram of KOfam annotations per gene call for the full set of metagenomes including those with shallow sequencing depth.*

– The visualizations are extremely data dense to the point that they are borderline noneffective. I am still not sure I understand every track/panel of Figure 1. Because of the visualization choices it is often not possible to know what the units are or what the column/rows represent and/or spot the points being made by the authors in text. In the ridge plots of Figure 3, if I correctly understand what has been plotted, it is difficult to think that these would be statistically significant as indicated.

*Response: We agree with the reviewer that our visualizations often pack a large amount of information. The ever-increasing size and complexity of datasets present significant challenges for anyone committed to transparent reporting of findings. Every data point shown in our figures are also available as reproducible data items. We now have updated our figure captions to clarify the meaning of individual tracks. The underlying data for the ridge plots in Figure 3 are indeed statistically significant as reported in the figure, in the text, as well as in our reproducible workflow.*

*We thank the reviewer again for their intriguing questions.*

Reviewer #2:

In this study, Andrea Watson et al investigated the association between metabolic modules of metagenomics and resilience in stressed gut environments, using fecal microbiota transplantation (FMT) experiment (2 donors and 10 recipients). The topic is of broad interest in this field. However, there are many major flaws in the study design, data analysis, and result interpretation. The overall conclusion is not supported by the current data (over concluded), and a more nuanced conclusion might be needed.

*Response: We thank the reviewer for their evaluation of our work and their comments. We appreciate their main concern, and we hope that our response, changes in the revised manuscript, and the additional data and insights we provide is sufficient to refute the claim of a flawed study design, or over-interpretation of our data.*

Specific comments:

– One of the major concerns is the small sample size (only 2 donors and 10 recipients). Reproducibility needs to be considered before making any meaningful conclusion. It is not clear why the authors only included a subset of individuals who participated in the randomized clinical trial.

*Response: The selection of donor and recipient samples were conducted by the three senior authors of this study (which includes Bana Jabri, a world-renowned immunologist who focuses on host-microbe interactions, and Thomas Louie, a world-renowned clinician who has been one of the pioneers of the clinical applications of FMT). We selected the donor and recipient samples from the larger clinical study Louie and his group conducted after a careful consideration of multiple critical factors that were not applicable to all participants of the clinical study. Briefly, we expected our sampling strategy to include (1) donors that contributed a large number of fecal samples over long periods of time (to maximize the number and quality of genomes from metagenomes AND to be able to address questions regarding the intrapersonal variability of the human microbiota if they were to arise), (2) donors whose feces were transplanted to the largest number of recipients (to be able to discuss the colonization dynamics of the same donor populations in different individuals accurately), and (3) recipients that were followed post-FMT the longest (to be able to follow donor population dynamics accurately). These were the characteristics that set our work apart from many others, which were confounded by short follow-up time of recipients, and/or large number of donor-recipient pairs that could not be used to address the questions we aimed to address in our work. The design of the study was unique, different from previous work, and was done with care.*

*Large cohorts do not automatically yield stronger insights, small cohorts do not automatically fail researchers. But the dangerously superficial focus on cohort size either forces researchers to use the limited resources they borrow from the public to design dull cross-sectional studies that generate shallow insights from a large number of individuals, or serve as gatekeepers to disseminate the same ideas from those who happen to have more resources. Reproducibility is indeed important, and we were able to test the hypotheses we generated using our cohort in a large collection of global metagenomes. To be clear, the data our study makes sense of is not small by any definition: there are 109 novel metagenomes in our study that exceed 7 billion sequences in size and resulted in more than 400 new microbial genomes that were benchmarked against an additional 1,000 publicly available gut metagenomes that originate from more than 10 independent studies across the globe. We used an additional 500 genomes from three distinct studies that cover multiple different diseases to scrutinize our hypothesis on metabolic competence. With the same resources we could indeed have designed a study with 10 FMT donors sampled at a single point, and 40 recipients that are sampled twice. While that study design may have passed the scrutiny of ‘sample size’ considerations, we would have not been able to make any of the observations we were able to make thanks to our longitudinal sampling and deep sequencing. Our study offers in-depth analyses of large amounts of sequences and benchmarks its insights by scaling those observations to the globe to support its conclusions and hypotheses. Regardless, our conclusions will certainly be scrutinized by ourselves and others in the long run.*

*Indeed, this unique cohort design enabled us to,*

*(1) Show the role of adaptive ecological processes in microbial colonization after FMT, which has not been shown previously. Previous studies gave credence to dose-dependence rather than adaptive processes, and our work is the first to demonstrate the substantial influence of adaptive processes compared to other factors. We achieve this level of clarity through genome-resolved metagenomics to reconstruct microbial genomes directly from donor metagenomes and with benchmarks that use more than 1,000 gut metagenomes from more than 10 independent studies.*

*(2) Observe that the adaptive ecological processes that allow for successful colonization post-FMT are parallel to those that shape microbial community composition in patients with IBD, independent of taxonomy. In particular, ‘metabolic independence’, as indicated by the presence of highly complete essential metabolic pathways, emerged as a key characteristic of successful microbes in our study and not only explained the microbial succession post-FMT, but also provided new insights into the gut microbial diversity in IBD patients. We confirmed this finding using two different IBDs (Crohn’s disease and pouchitis) and genomes reconstructed from three additional, independent studies.*

*(3) Shed light onto one of the most mysterious conundrums of gut microbial ecology: Why are widespread, yet typically low-abundance members of healthy gut environments often dominant in IBD gut metagenomes? Our findings indicate that resilience conferred by metabolic competency is the key for success under challenging environmental conditions, such as post-FMT or as a result of IBD. The novel answer our study offers to this essential question is based upon an exceedingly comprehensive orchestration of state-of-the-art 'omics strategies, FMT experiments, global gut metagenomes, and metabolic reconstruction.*

*As the reviewer points out, these are broadly interesting to the community who ponder about these questions, and it is critical to communicate these ideas in a timely fashion. Overall, we strongly disagree with the reviewer that there were many major flaws in the study design, data analysis, and result interpretation.*

– Importantly, the host factors (eg. age, sex, race, etc.) and the dietary factors, which could greatly affect the gut microbiome composition, were not considered at all in this study.

*Response: The reviewer highlights one of the strengths of our study as one of its weaknesses.*

*It is true that the interpersonal variation across human gut microbiota that is manifested due to differences between host as well as environmental factors has been one of the most significant challenges in human gut microbial ecology to identify robust microbial markers for health or disease states. Our study shows that by changing our focus from a taxonomic framework to one that considers metabolic potential to characterize competencies of microbes using an appropriate experimental design, it is possible to observe trends that are predictive of microbial success and prevalence in stress regardless of phylogeny or host factors.*

*To identify such metabolic competencies that were associated with survival in stressful conditions, we indeed used a cohort of donors and recipients that were not homogenized based on genetics, ethnicity, gender, or lifestyles. Most microbial ecologists would agree that microbial markers proposed to shed light on ecosystem states across cohorts must be resilient to host and dietary factors. Our data suggests that the extent of metabolic independence of a given microbial genome can be that strong marker for the colonization success and survival of a given microbial organism in stressful environments, whether it is post-FMT colonization of recipients, or survival in IBD, and shows that this signal is not limited to our study.*

*Here, we would like to include in our response additional insights from a study in-progress that continued to build on the observations made in the current study. In the current study, we used genome-resolved metagenomics in combination with FMT as a model to study determinants of colonization success, which enabled us to identify a set of metabolic modules responsible for the biosynthesis of essential nutrients as a marker of successful colonizers. We then hypothesized in the current study that, if successful colonization is a predictor of survival in stressful conditions, then the same set of metabolic modules should be enriched in microbial genomes reconstructed from individuals who have IBD, compared to individuals who are healthy. In the current study we were able to confirm that it indeed seems to be the case based on the publicly available metagenome-assembled genomes we obtained from other studies that reconstructed microbial genomes from healthy individuals and patients diagnosed with IBD. Given the thousands of metagenomes in public databases, we could further test this hypothesis in much larger cohorts. However, being able to accurately quantify the extent of metabolic independence in a given metagenome without reconstructing genomes posed a significant technical challenge. So in our study in-progress, we implemented a new software framework and a machine learning approach to scale up observations in this current study to a much larger cohort. Our new results, which take into account almost 3,000 metagenomes, indicate that a good fraction of metabolic modules we have identified in this current study has significantly higher ‘per-population copy number’ in metagenomes from individuals diagnosed with IBD compared to those from healthy individuals. Furthermore, we were able to implement a classifier based on these new findings, which was able to identify healthy individuals and those diagnosed with IBD at high accuracy and sensitivity. The classification model has an AUROC of 0.832. AUROC, the ‘area under the receiver operating characteristic’, is a commonly-used metric to evaluate the classification performance of machine learning models where an AUROC of 0.5 suggests zero discrimination power for the model, 0.7 to 0.8 is considered acceptable, and 0.8 to 0.9 is considered excellent. Overall, the enrichment of certain metabolic modules revealed in the current study continues to be a strong signal to distinguish microbial features observed in individuals who are healthy or IBD without reconstructing genomes from metagenomes and independent of host and dietary factors. The figure below summarizes the differential enrichment of the metabolic molecules in IBD and healthy cohorts (where each dot represents the median per-population copy number of a given metabolic module after processing 2,893 publicly-available gut metagenomes from 13 different studies):*

*On the one hand, this follow-up work that builds on the concepts our current work introduces is not yet published, thus we anticipate that it is difficult to take it into consideration to review this current study. On the other hand, we hope that the reviewer and the editor can at least relieve themselves from concerns of the validity, interest, and generalizability of our observations here, and/or concerns related to the cohort size and data analyses that shaped our conclusions.*

– Another concern is the determination of MAGs representing "good" and "poor" colonizers for metabolic enrichment analysis. The authors classified MAGs as "poor colonizers" as those that, 'in at least 3 recipients", were detected in the donor sample used for FMT but were not detected in the recipient at least 7 days post-FMT. Due to the small sample size of the study, this determination method (i.e. "in at least 3 recipients",) could be problematic.

*Response: The reviewer would have been absolutely correct if our approach to classify our genomes as good or poor colonizers was limited to the outcomes of FMT alone, but in fact the initial observations of colonization success after FMT is benchmarked against a large number of publicly available metagenomes. Our final determination of poor or good colonizers also considers their distribution patterns across over 1,000 publicly available metagenomes. We outline in our manuscript, “populations identified as poor colonizers were less prevalent than good colonizers in each of the 17 different countries we queried. In countries including the United States, Canada, Austria, China, England, and Australia, microbial populations identified as good colonizers occurred in 5 times more people than poor colonizers in the same country (Figure 1, Supplementary Table 3)”. The strong support from global gut metagenomes speaks to the accuracy of our approach.*

*Additionally, our longitudinal study design allowed us to confidently classify individual colonization/non-colonization events. Our criteria for colonization took into account the possibility of lowly abundant populations or fluctuations in population relative abundance resulting in some populations being only sporadically detectable in recipient samples. Unlike other studies with fewer longitudinal samples per subject, our study design minimized the chance that we will miss a colonization event due to an insufficient number of post-FMT samples in which to detect a colonizing population. Similarly, our longitudinal sampling allows us to confidently designate non-colonization events, by ensuring that we do not falsely classify transient populations only present in the recipient gut less than one week post-FMT as colonizers.*

– Page 11, Line 265. "enrichment analysis between good and poor colonizers revealed 33 metabolic modules that were enriched in good colonizers". How many metabolic modules were included in the analyses in total? This information is important for enrichment test, which needs to be described in the main text.

*Response: We thank the reviewer for pointing this out. We have updated our manuscript with the following sentence to indicate the total number of modules considered: “Our enrichment analysis between good and poor colonizers revealed 33 metabolic modules (out of 443 total in the KEGG MODULE database)”.*

– Since this study has many analyses, thus multiple testing correction is needed.

*Response: The only statistical analysis in which we conduct many simultaneous significance tests is the enrichment analysis for metabolic modules. Multiple testing correction is inherent to the calculation of q-values in our enrichment analysis and is taken care of by the enrichment analysis algorithm cited. From our methods section: “We considered any function or metabolic module with a q-value less than 0.05 to be ‘enriched’ in its associated group if it was also at least 75% complete and present in at least 50% of the group members.”*

– The author defined 'high metabolic independence' (HMI) as genomic evidence for a population's ability to synthesize essential compounds. Some more detailed criteria are needed for this definition.

*Response: We thank the reviewer for pointing this out. Their comment helped us recognize that a more specific and reproducible criteria would be beneficial to the community to test or apply ideas proposed in our study to other datasets.*

*In our study we hypothesize that the metabolic independence of a given organism is a function of the sum of the completeness scores of the metabolic pathways we determined to be enriched in good colonizers. We consider a given genome to represent a population with high metabolic independence if the sum of these subsets of metabolic modules is above 20. The figure below visualizes the distribution of these scores across genomes with high and low colonization performance, where 20 as a threshold seems to be a fair starting point for downstream analyses:*

*For further reproducibility and downstream practical applications of this logic, we implemented a Python script that can perform HMI/LMI classification on any genome, which is now a part of the anvi’o open-source software ecosystem:*

*https://anvio.org/m/anvi-script-estimate-metabolic-independence*

*We have also clarified our definition of HMI in the text, which now reads: “For the remainder of our study, the term ‘high metabolic independence’ (HMI) describes genomic evidence for a population's metabolic capacity encoded by 33 metabolic modules described above. For a genome to have high metabolic independence, the sum of its completeness scores for these 33 modules should be at least 20 (see Methods).”*

– Figure 1. For each of the metagenome-assembled genomes, the species names need to be labeled. The current label like "00052" do not provide any meaningful information. In addition, it would be helpful to also highlight some of the known predominant bacteria taxa, such as Provotella and Bacteroides. What's the coverage for each of the metagenome-assembled genome? This kind of key information needs to be shown in Figure 1.

*Response: Those unique labels match to entries in our supplementary tables, such as Supplementary Table 2, which includes a large amount of information regarding the MAGs, including their length, number of contigs, N50, GC-content, percent completion and redundancy based on single-copy core gene presence/absence frequencies, their coverage and detection in global metagenomes, as well as taxonomy for all levels, including species. Our work does not have a particular focus on taxonomy, thus adding this information to an already complex figure will lower its readability further. Furthermore, many of our genomes do not have any species-level taxonomic annotation beyond GTDB-assigned codes, such as CAG-273, TF01-11, UBA11774, etc, which is the reality of genome-resolved metagenomics and the current state of taxonomy.*

– It would be interesting to explore why 90% of the metagenome-assembled genomes in Figure 1 belonged to "Firmcutes" phylum. What happened to the other known predominant phylum, such as "Bacteroidetes" and "Proteobacteria"?

*Response: The number of assembled MAGs belonging to a particular phylum does not necessarily indicate a higher abundance of that phylum. That being said, the numerous Firmicutes MAGs we assembled does reflect a predominance of that phylum in our donor samples (with a median relative abundance of 67% Firmicutes across all donor samples). The high percentage of short reads recruited by our MAGs (an average of 80% across all donor samples) demonstrates that these MAGs represent the microbial populations present in the donor guts well.*

– Supplementary Figure 2 and 3. The beta-diversity analyses were performed on genus level. This resolution is not good enough, especially for metagenomics sequencing. Strain level analyses is needed, since this study have already assembled microbial genomes. In addition, I would suggest to use the phylogenetic based matrix, such as weighted UniFrac distance.

*Response: The only beta-diversity analysis in our study was performed to show that there was a dramatic shift in recipient taxonomic profiles after FMT, which is a widely documented hallmark of this procedure. A higher resolution may have been necessary if there was no change between before- and after-FMT microbial community composition in recipients at the genus level, but it is not the case. Given the context of our beta-diversity analyses, a higher taxonomic level or a phylogenetically-informed distance estimate has no additional value to make this unsurprising and clear point.*

*We thank the editor and our reviewers again for their evaluation of this work.*

**Second round of review**

**Reviewer 1**

I thank the authors for their thoughtful responses. I would propose only that the reviewer figure pertaining to potential annotation bias be included as a supplemental figure and that the others include a brief discussion of this potential in the manuscript.

**Reviewer 2**

Many of major concerns I mentioned in the previous comments are not addressed. Further revision is needed.

Specific comments:

- The issue of small sample size (i.e. 2 donors and 10 recipients). The major concern for this study is that, the small sample size (i.e.2 donors and 10 recipients) may not be truly “representative” of the population from which it is drawn. The risk of excluding important population characteristics increases for a small sample size like this. The risk of selection bias where there is a concentration of certain characteristics in your sample also increases.

Please note that, no matter how deep you sequenced the samples from one single individual, the bias is still there. The conclusions were overstated based on data from the current small sample size study. Sometimes the results could be even misleading. A more nuanced conclusion is required.

- As I mentioned earlier, it is not clear why the authors only include a subset of individuals who participated in the randomized clinical trial. The criteria of sample selection should be described in the “methods” section in details. Why you selected these 10 recipients in the randomized clinical trial?

- The host factors (eg. age, sex, race, etc.) and the dietary factors, which could greatly affect the gut microbiome composition, were not appropriately adjusted or controlled by statistical or experimental methods. Although this study included some of the publicly available metagenome-assembled genomes, due to the heterogeneity of the host, it could be misleading to compare these data without adjusting for host factors. Also, this study did not explain to what extent the host factors (eg. age, sex, race, etc.) and the dietary factors might affect the findings. It would be helpful to clarify whether these results could be explained by the host factors. It is important to include the details of methods to control for the confounding variables in the method section.

- Figure 1. For each of the metagenome-assembled genomes, the current label like “00052” do not provide any meaningful information to readers. I would suggest to directly label the species name (at least for those identified species with known name) rather than a meaningless ID number, and also highlight the predominant bacteria taxa, and those assembled genomes with average coverage depth >10X.

- As mentioned previously, “It would be interesting to explore why 90% of the metagenome-assembled genomes in Figure 1 belonged to "Firmicutes" phylum. What happened to the other known predominant phylum, such as "Bacteroidetes" and "Proteobacteria"?” What was the potential reason? The current “response” did not answer this question. It would be good to discuss the potential reasons in the Discussion section. In addition, did the authors apply de novo assembly methods for any of the metagenome-assembled genomes? Or just simplify did a reference mapping?

- As mentioned previously, since this study has already assembled many microbial genomes, it would be better to incorporate the phylogenetic information in this analysis to provide more reliable results.

**Authors Response**

**Point-by-point responses to the reviewers’ comments:**

Reviewer #1:

I thank the authors for their thoughtful responses. I would propose only that the reviewer figure pertaining to potential annotation bias be included as a supplemental figure and that the others include a brief discussion of this potential in the manuscript.

*Response: We thank the reviewer for their suggestion. Our revised manuscript includes multiple changes to communicate this observation. First, we extended our remarks on limitations of our study by adding the following sentence,*

*Yet, our study is limited to well-known metabolic pathways – which, given the extent of the unknown coding space in microbial genomes (Vanni et al. 2020), are likely far from complete – as well as by our ability to recognize gene function, which is determined by the sequences described in public databases that favor well-studied microbial organisms (Supplementary Information).*

*And created a new section in our Supplementary Information file with the following subtitle, text, and figure, which allowed us to present these data with a more complete context rather than a passing comment:*

***Considerations of Annotation Bias That Reduce the Number of Genes with Functional Annotations for Microbial Populations that Typically Occur in Healthy Individuals***

*Our study, and most others in microbiology and genomics, depend on accurate functional annotations of microbial genes whether they are found in genomes or metagenomes. The efficacy of annotation is not uniform across different taxa and environments since the representation of microbial genomes (or our desire to extensively characterize them) are not uniform throughout the tree of life or across ecosystems. Arguably, the human gut is one of the better studied ecosystems since microbial organisms found in this habitat can dramatically influence human health. Yet, even for this relatively well-studied environment, our ability to annotate genes with known functions is apparently not quite uniform across cohorts of individuals. Here we investigated the proportion of genes with KOfam annotations in a large set of publicly-available gut metagenomes and found that, for any given metagenome, the ratio of the number of genes that were annotated by KOfams in the assembly to the total number of genes recovered were lower in samples from healthy people compared to those who were diagnosed with inflammatory bowel disease, at least in deeply-sequenced metagenomes with adequate coverage of low-abundance populations (Figure SI2). Given the much higher diversity of the gut microbiome in homeostasis and the presence of many rare taxa with poorly cultured representatives, this outcome is not entirely surprising. However, it is difficult to identify the extent to which the observed reduction in metabolic potential in our study is due to missing annotations, even though there is substantial evidence from the FMT experiment that LMI genomes have distinct ecological behavior beyond their functional annotations. The significant reduction in genome sizes and therefore the overall reduced functional repertoire of LMI populations that likely hinders cultivation efforts, is also likely one of the reasons that contributes to their depletion in the IBD gut. Therefore, while this bias does not necessarily affect our hypotheses, it does warrant future studies to investigate the underpinnings of this bias and its implications on our ability to understand the microbial ecology of the human gut environment.*

*Figure SI2. Histogram of number of KOfam annotations per gene call in publicly-available gut metagenomes from healthy people and from people with inflammatory bowel disease (IBD). Panel a) shows data for 330 deeply-sequenced samples with at least 25 million sequencing reads. Panel b) shows data for all 2,893 samples, including non-IBD controls.*

*We are thankful for the suggestion that allowed us to think more about this anomaly with data analyses and helped us push our research further.*

Reviewer #2:

Many of major concerns I mentioned in the previous comments are not addressed. Further revision is needed.

*Response: We thank the reviewer for their time and effort. Many of the outstanding comments by Reviewer #2 are identical to their original concerns, which we responded to in our previous revision and response to reviewers. We strongly believe that our responses, changes in the revised manuscript, and the additional data and insights we provide are sufficient to refute the Reviewer’s claim of ‘flawed study design’, or ‘over interpretation’.*

– The issue of small sample size (i.e. 2 donors and 10 recipients). The major concern for this study is that, the small sample size (i.e.2 donors and 10 recipients) may not be truly “representative” of the population from which it is drawn. The risk of excluding important population characteristics increases for a small sample size like this. The risk of selection bias where there is a concentration of certain characteristics in your sample also increases. Please note that, no matter how deep you sequenced the samples from one single individual, the bias is still there. The conclusions were overstated based on data from the current small sample size study. Sometimes the results could be even misleading. A more nuanced conclusion is required.

*Response: The reviewer once again brings up “the issue of sample size”, which we discussed in length in our response to reviewers. Overall, the reviewer suggests that results from a small sample size can be misleading, and more nuance in the conclusion is required. ‘Results can be misleading’ is an accurate remark, although, with all due respect, not a useful one that we can act upon. The sample size is not a secret and this information along with the study design is abundantly clear at the beginning of our Results section. Our conclusions offer a hypothesis after tremendous amounts of data analyses that make use of thousands of metagenomes.*

*Nevertheless, we removed the following sentence from our study:*

*Research into the development of microbial therapeutics to reinstate homeostasis is continuously intensifying (Jimenez et al., 2019) and the benefits of such treatments will critically depend on successful engraftment of individual populations. Thus, considering their degree of metabolic independence may prove crucial.*

*Since it was speculative and beyond the scope of our work.*

– As I mentioned earlier, it is not clear why the authors only include a subset of individuals who participated in the randomized clinical trial. The criteria of sample selection should be described in the “methods” section in details. Why you selected these 10 recipients in the randomized clinical trial?

*Response: We now have updated our methods section with the following sentence to detail the sample selection criteria:*

***Sample collection and storage.*** *We selected our samples from a subset of individuals who participated in a randomized clinical trial (Kao et al. 2017). Our selection criteria took into consideration multiple factors that were not applicable to all participants of the clinical study. Briefly, we aimed to identify (1) donors that contributed a large number of fecal samples over long periods of time (to maximize the number and quality of genomes from metagenomes and to be able to identify the extent of intrapersonal variability of the microbiota and its potential impact on our results), (2) donors whose feces were transplanted to the largest number of recipients (to be able to discuss the colonization dynamics of the same donor populations in different individuals accurately), (3) multiple recipients for each donor that received FMT via different methods, such as colonoscopy versus pills (to be able to better understand the generalizability of our downstream observations independent of the delivery method), and (4) recipients that were followed the longest period of time after FMT (to be able to follow donor population dynamics accurately). We did not consider factors that may impact the microbial community composition (such as age, gender, or diet) to homogenize the recipient cohort to observe overarching microbial patterns after FMT that are beyond environmental factors dictated by the host. Based on these criteria we identified two donors (DA and DB), and 5 FMT recipients for each donor. (...)*

*We thank the reviewer for insisting on this point. We believe it will be helpful to readers to have a better idea about the priorities of this study.*

– The host factors (eg. age, sex, race, etc.) and the dietary factors, which could greatly affect the gut microbiome composition, were not appropriately adjusted or controlled by statistical or experimental methods. Although this study included some of the publicly available metagenome-assembled genomes, due to the heterogeneity of the host, it could be misleading to compare these data without adjusting for host factors. Also, this study did not explain to what extent the host factors (eg. age, sex, race, etc.) and the dietary factors might affect the findings. It would be helpful to clarify whether these results could be explained by the host factors. It is important to include the details of methods to control for the confounding variables in the method section

*Response: As we mentioned in our previous response, the heterogeneity of donors and recipients is a strength of this study to learn something that is beyond environmental factors dictated by the host (such as age, sex, or diet). We updated our methods section (see above) to clarify that such factors don’t need adjustment due to the study design and priorities.*

– Figure 1. For each of the metagenome-assembled genomes, the current label like “00052” do not provide any meaningful information to readers. I would suggest to directly label the species name (at least for those identified species with known name) rather than a meaningless ID number, and also highlight the predominant bacteria taxa, and those assembled genomes with average coverage depth >10X.

*Response: The current labels do not mean to provide any meaningful information to the readers, but they match to entries in our supplementary tables, such as Supplementary Table 2, which includes a large amount of information regarding the MAGs, including (1) their length, (2) number of contigs, (3) N50, (4) GC-content, (5) percent completion and redundancy based on single-copy core gene presence/absence frequencies, (6) their coverage and detection in global metagenomes, as well as (7) taxonomy for all levels, including species. Our work does not have a particular focus on taxonomy, thus adding this information to an already complex figure will lower its readability further. Furthermore, many of our genomes do not have any species-level taxonomic annotation beyond GTDB-assigned codes, such as CAG-273, TF01-11, UBA11774, etc, which is the reality of genome-resolved metagenomics and the current state of taxonomy. We do not see the significance of ‘10X’, and the utility of highlighting genomes that have higher coverage than this arbitrary number.*

– As mentioned previously, “It would be interesting to explore why 90% of the metagenome-assembled genomes in Figure 1 belonged to "Firmicutes" phylum. What happened to the other known predominant phylum, such as "Bacteroidetes" and "Proteobacteria"?” What was the potential reason? The current “response” did not answer this question. It would be good to discuss the potential reasons in the Discussion section. In addition, did the authors apply de novo assembly methods for any of the metagenome-assembled genomes? Or just simply did a reference mapping?

*Response: We are surprised by the reviewer question “did the authors apply de novo assembly methods for any of the metagenome-assembled genomes? Or just simply did a reference mapping?” as the de novo reconstruction of genomes directly from donor metagenomes is one of the core strengths of our work. We indeed did not simply do a reference mapping.*

*Regarding the large number of Firmicutes genomes, we conducted an analysis to communicate the fact that it is not an anomaly, but an expected composition of the gut microbiota of industrialized human populations. For our large-scale analysis to investigate this point, we first identified microbes that are commonly detected in the human gut by analyzing 19,226 reference genomes from the Genome Taxonomy Database (GTDB) that belonged to three major phyla take make up over 99% of the gut microbiota: Proteobacteria, Firmicutes, and Bacteroidetes. We first performed a read recruitment analysis and removed any genome from this collection whose ribosomal protein sequences had insufficient coverage in metagenomes from the Human Microbiome Project. This gave us a list of 836 genomes that belonged to organisms that were associated with the human gut environment. We then performed a read recruitment analysis on these genomes using 330 publicly-available gut metagenomes from healthy people (n=229) and from people who were diagnosed with inflammatory bowel disease (n = 101). We identified gut microbes as those for which the genome sequence was at least 50% covered by at least one sequencing read in over 2% (n > 6) of these samples. Using this strategy, we obtained a final set of 338 gut microbes for which the distribution of phyla closely resembled the one seen in the current study. The vast majority of these genomes (n=258, 76.3% of the final set) were Firmicutes, meanwhile 60 (17.8%) were Bacteroidetes, and 20 (5.9%) were Proteobacteria. The figure below demonstrates the phylogeny of these genomes, and shows the numerical dominance of Firmicutes and its extensive diversity compared to Bacteroidetes in the global human gut of industrialized populations. Note that the metagenomes used for read recruitment were all deeply sequenced (> 25 million sequencing reads) and should have sufficient coverage of even low-abundance populations. Thus, we have been able to confirm on a larger scale that it is not unusual to see the vast majority of gut microbes belong to Firmicutes rather than the other ‘predominant’ phyla:*

– As mentioned previously, since this study has already assembled many microbial genomes, it would be better to incorporate the phylogenetic information in this analysis to provide more reliable results.

*Response: The phylogenetic information regarding our genomes are already in our figures and Supplementary Tables.*

**Third round of review**

**Reviewer 1**

The authors have more than adequately addressed my concerns.

**Reviewer 2**

No further comments.
